# Supplementary material for: A longitudinal study to assess the frequency and cost of antivascular endothelial therapy, and inequalities in access, in England between 2005 and 2015
Source: BMJ Open. 2017 Oct 22;7(10):e018289. doi: 10.1136/bmjopen-2017-018289 (PMC5665286; doi:10.1136/bmjopen-2017-018289)
Supplement: Author's manuscript [file bmjopen-2017-018289.draft_revisions.pdf]

BMJ Open is committed to open peer review. As part of this commitment we make the peer review history of every article we publish publicly available.

When an article is published we post the peer reviewers' comments and the authors' responses online. We also post the versions of the paper that were used during peer review. These are the versions that the peer review comments apply to.

The versions of the paper that follow are the versions that were submitted during the peer review process. They are not the versions of record or the final published versions. They should not be cited or distributed as the published version of this manuscript.

BMJ Open is an open access journal and the full, final, typeset and author-corrected version of record of the manuscript is available on our site with no access controls, subscription charges or pay-per-view fees (http://bmjopen.bmj.com).

If you have any questions on BMJ Open's open peer review process please email editorial.bmjopen@bmj.com

BMJ Open

The cost of turning a blind eye to unlicensed medicines: the impact of NICE recommendations on patient access to anti-VEGF therapy

Journal:	*BMJ Open*
Manuscript ID	bmjopen-2017-018289
Article Type:	Research
Date Submitted by the Author:	16-Jun-2017

Complete List of Authors:	Hollingworth, William; University of Bristol, School of Social and Community Medicine<br>Jones, Tim; NIHR CLAHRC West,<br>Reeves, Barnaby; University of Bristol, Bristol Heart Institute<br>Peto, Tunde; Queen's University Belfast Faculty of Medicine Health and Life Sciences

**Primary Subject Heading**:	Health policy
Secondary Subject Heading:	Health economics, Ophthalmology, Evidence based practice, Public health
Keywords:	Vascular Endothelial Growth Factor A, Drug Costs, macular degeneration, macular edema, England, Geography

SCHOLARONE™
Manuscripts

For peer review only

Title: The cost of turning a blind eye to unlicensed medicines: the impact of NICE recommendations on patient access to anti-VEGF therapy

Authors: William Hollingworth[1], Tim Jones[2], Barnaby C Reeves[3], Tunde Peto[4]

1 Professor of Health Economics, School of Social and Community Medicine, University of Bristol, Bristol, BS8 2PS, United Kingdom

2 Senior Research Associate, NIHR CLAHRC West, University Hospitals Bristol NHS Foundation Trust, Bristol, BS1 2NT United Kingdom

3 Professorial Research Fellow in Health Services Research, Clinical Trials Evaluation Unit, School of Clinical Sciences, University of Bristol, Bristol, BS2 8HW, United Kingdom

4 Clinical Professor, School of Medicine, Dentistry and Biomedical Sciences, Queen's University Belfast, Belfast, BT9 7BL, United Kingdom

Corresponding author:

William Hollingworth
Professor of Health Economics
Health Economics at Bristol (HEB)
School of Social and Community Medicine
University of Bristol
Canynge Hall
39 Whatley Road
Bristol, BS8 2PS
United Kingdom

Tel: ++44 (0)117 9287355
william.hollingworth@bristol.ac.uk

Word count [3,564]

Keywords:
Vascular Endothelial Growth Factor A; Drug Costs; macular degeneration; macular edema; England; socioeconomic factors; geography

For peer review only - http://bmjopen.bmj.com/site/about/guidelines.xhtml

Abstract [310 words]

Objectives: High cost anti-VEGF medicines for eye disorders challenge ophthalmologists and policymakers to provide fair access for patients while minimising costs. We describe the growth in the use and costs of these medicines and measure inequalities in access.

Design: Longitudinal study using hospital episode statistics (2005/6 to 2014/15) and hospital prescribing cost reports (2008/9 to 2015/16). We used Poisson regression to estimate standardised rates and explore temporal and geographic variations.

Setting: NHS care in England.

Population: Patients receiving anti-VEGF injections for age-related macular degeneration, diabetic macular oedema and other eye disorders.

Interventions: Higher cost drugs (ranibizumab or aflibercept) recommended by NICE or lower cost drug (bevacizumab) not licensed for eye disorders.

Main outcome measures: National procedure rates and variation between and within clinical commissioning groups (CCGs). Cost of ranibizumab and aflibercept prescribing.

Results: Injection procedures increased by 215% between 2010/11 and 2014/15. In 2014/15 there were 388,031 procedures (714 per 100,000). There is no evidence that the dramatic growth in rates is slowing down. Since 2010/11 the estimated cost of ranibizumab and aflibercept increased by 247% to £447 million in 2015/16, equivalent to the entire annual budget of a CCG. There are large inequalities in access; in 2014/15 procedure rates in a 'high use' CCG were 9.08 times higher than in a 'low use' CCG. In the South West of England there was twofold variation in injections per patient per year (range 2.9 to 5.9).

Conclusions: The huge cost of anti-VEGF therapy increasingly affects the ability of the NHS to provide care for other patients. There is a lack of political and regulatory support for clinicians to use bevacizumab, which evidence suggests is more cost-effective. NICE aims to ensure that patients have equal access to cost-effective care. In this instance, it has not been able to achieve either goal.

For peer review only - http://bmjopen.bmj.com/site/about/guidelines.xhtml

Strengths and limitations

- Our study summarises new data on nationwide access to effective, but high cost ophthalmology drugs.
- Using population denominators allows us to standardise our analysis for factors, such as age and socio-economic deprivation, which will affect the need for care.
- The lack of a specific anti-VEGF drug code in HES is a limitation, however we observed similar temporal trends in two independent data sources.
- The confidential nature of drug discounts mean that our estimate of drug costs will be too high if commissioners have negotiated substantial discounts.
- Conversely, the exclusion of administration and monitoring costs make our estimates conservative.

What this paper adds

What is already known

- NICE recommended two expensive drugs (ranibizumab and aflibercept) for the treatment of conditions affecting the eye, despite the availability of a more cost-effective, but unlicensed, alternative (bevacizumab).
- Healthcare commissioners struggle to provide fair access to these drugs while minimising costs.

What this study adds

- Our study demonstrates a 215% increase in injection procedures between 2010/11 and 2014/15 with large variations in procedure rates across England.
- In 2015/16, the NHS spent an estimated £447 million on drug costs alone, equivalent to the entire annual budget of a large CCG.
- In this instance, NICE has not achieved equal access to cost-effective care.

For peer review only

For peer review only - http://bmjopen.bmj.com/site/about/guidelines.xhtml

Introduction

Clinical commissioning groups (CCGs) in England are responsible for commissioning healthcare for a geographically defined local population. On average, CCGs cover a population of 270,000 and have a budget of £300 million to commission services including elective and unplanned hospital care, community and mental health services. While CCGs have a degree of autonomy in the care they commission, they must work within national constraints. In particular, CCGs must commission new interventions which have been evaluated to be cost-effective by the National Institute for Health and Care Excellence (NICE) technology appraisal process. This presents a challenge as many of these interventions are expensive. A balance is needed in order to provide fair access for patients eligible for the new interventions without disadvantaging other patients using existing health services.

One recent challenge for CCGs is to commission anti vascular endothelial growth factor (anti-VEGF) medicines for patients with a range of eye conditions. Anti-VEGF intravitreal injections reduce new blood vessel growth and swelling [1]. Two drugs (ranibizumab and aflibercept) are licensed for use in neovascular age-related macular degeneration (nAMD), macular oedema due to diabetes (DMO) or retinal vein occlusion (RVO), and myopic choroidal neovascularisation (MCNV). Ranibizumab was first recommended by NICE as cost-effective for use in nAMD in 2008 [2]. After an initial rejection in 2011, NICE also recommended the use of ranibizumab for DMO in 2013 based on new evidence and an undisclosed discount on price offered by the manufacturer [3]. Subsequent NICE recommendations in other eye conditions and for aflibercept have followed (Table 1). Ranibizumab and aflibercept have an NHS indicative price in the region of £550 and £800 per injection, respectively [4]. The high prevalence of these conditions, the indefinite need for multiple injections per patient, and the associated procedure and monitoring costs mean that economic burden is substantial.

In reaching their recommendations, NICE did not compare ranibizumab or aflibercept with a pre-existing drug, bevacizumab. Bevacizumab also inhibits VEGF and is closely related to ranibizumab; it

For peer review only - http://bmjopen.bmj.com/site/about/guidelines.xhtml

is licensed as a treatment for certain types of cancer but not in ophthalmology. Bevacizumab can be divided into aliquots of smaller doses for ophthalmic therapy and is a much less costly alternative (approximately £50 to £100 per injection) [5]. Meta-analysis has demonstrated that bevacizumab and ranibizumab result in similar visual acuity outcomes for patients with nAMD [6]. The IVAN randomised trial of ranibizumab versus bevacizumab in nAMD demonstrated that ranibizumab produced little or no gain in quality-adjusted life years (QALYs) despite being substantially more expensive [7]. Individual patient data meta-analysis indicates that the risk of serious adverse events with ranibizumab and bevacizumab are similar in nAMD [8]. In DMO, the DRCR.net three-arm randomised trial found that aflibercept and ranibizumab were not cost-effective relative to bevacizumab despite evidence that aflibercept resulted in better visual outcomes at 1 year [9]. In RVO, the SCORE2 randomised trial concluded that bevacizumab was non-inferior to aflibercept for visual acuity after 6 months of treatment [10]. In 2014, the Royal College of Ophthalmologists and others called on UK health regulators, including NICE, to urgently review the use of bevacizumab in nAMD [11]. However, in 2015 the Department of Health declined to support clinicians or commissioners using bevacizumab for nAMD, considering unlicensed use "on ground of cost alone" as potentially illegal under EU law [12], despite political support for the use of bevacizumab for ophthalmic indications in other EU countries such as Italy [13]. The remit of the forthcoming NICE nAMD clinical guidelines preclude making recommendations about bevacizumab [14].

The emergence of anti-VEGF medicines provides ophthalmologists and CCGs with a clinical and economic dilemma in both providing fair access for eligible patients to drugs recommended by NICE while minimising the opportunity costs for other members of the population. In this study, we describe the growth in the use and cost of anti-VEGF intravitreal injections in the English NHS since 2005/06. We quantify the variation in patient access to anti-VEGF therapy across England and explore the potential causes of inequality of access within the South West region of England.

For peer review only

For peer review only - http://bmjopen.bmj.com/site/about/guidelines.xhtml

Methods

IDENTIFYING ANTI-VEGF INJECTIONS

We used the HES admitted patient care (APC) and outpatient (OP) datasets to identify anti-VEGF injections. Anti-VEGF injections are performed in outpatient treatment rooms, but, in earlier years, were often coded in the APC dataset as day case procedures. HES is a routinely collected dataset that records all episodes of care provided to NHS funded and privately insured patients admitted (day case or inpatient) to NHS hospitals or seen at an NHS outpatient clinic [15]. HES also includes data on NHS-funded patients treated in independent sector hospitals. We extracted anonymised individual episode records on all episodes contained in the HES APC (2005/6 to 2014/15) and OP (2010/11 to 2014/15) datasets. Up to 24 clinical procedures per episode may be recorded using Office of Population, Censuses and Surveys (OPCS) (fourth revision) codes.

Anti-VEGF injection procedures are not coded consistently. The majority of healthcare providers used the code 'injection into vitreous body, not elsewhere classified [C79.4]'. In preliminary exploration of HES data we looked for negative correlations between this procedure code and other ophthalmology procedure codes to identify potential alternate procedure codes. This identified that a substantial minority used a newer code 'Injection of therapeutic substance into posterior segment of eye, not elsewhere classified [C89.3]'. HES does not record the specific drug injected into the eye; however, since 2009 an OPCS code for high cost subfoveal choroidal neovascularisation drugs Band 1 [X93.1] has been in use. This code may be used for aflibercept, bevacizumab, ranibizumab, and two other drugs rarely used in intravitreal injections, pegaptanib and verteporfin. We excluded any procedures using non anti-VEGF drugs (X93.2, X93.3).

In 2014/15, 73.3% (284,490/388,031) of episodes including an eye injection procedure also coded a high cost subfoveal choroidal neovascularisation drug. However, some hospitals performing hundreds of intravitreal injections each year never use this high cost drug code. Conversely, 96%

For peer review only - http://bmjopen.bmj.com/site/about/guidelines.xhtml

(284,490/296,129) of episodes coding a high cost subfoveal choroidal neovascularisation drug also included an eye injection procedure (Figure 1). In our analysis, we assumed that procedures coded as C79.4 or C89.3 were anti-VEGF injections. We explore the importance of this assumption in our discussion.

A minority of patients receive bilateral injections on the same day, which may be coded using the supplemental 'bilateral operation' [Z94.1] OPCS code. When a patient had a bilateral procedure coded, we counted this as two injection procedures. When a patient had two injection procedures coded on the same day without any supplemental bilateral code, we assumed that this also represented bilateral procedures. If patients had more than two injection procedures coded on the same day, we excluded the 'excess' procedures as probable coding error duplicates (<0.03% of all procedures).

We used ICD-10 diagnosis codes to identify patients with diabetes with ophthalmic complications (E10.3, E11.3, E12.3, E13.3, or E14.3) or diabetic retinopathy (H36.0) or with a primary code indicating “other specified retinal disorders” (H35.8) combined with secondary codes indicating diabetes with ophthalmic complications as above; nAMD (H35.3); RVO (H34.8); and MCNV (H44.2) respectively. Diagnosis codes are well recorded for procedures in the HES APC dataset, however in the HES OP dataset diagnosis codes are often missing. Therefore, we limited our analysis of diagnostic indications to procedures recorded in the HES APC dataset.

ESTIMATING PROCEDURE RATES

National trends over time were estimated using directly standardised procedure rates [16] (per 100,000 population), with the population of England in 2014 as our standard population. We first summed the number of anti-VEGF injections, grouped by sex, quintiles of age, and financial year. These procedure counts were used to calculate annual age-sex-specific rates, by dividing by the appropriate age-sex-specific mid-year populations of England [17] (e.g. for the 2012/13 financial

For peer review only

For peer review only - http://bmjopen.bmj.com/site/about/guidelines.xhtml

year, the mid-2012 populations were used). We weighted the annual age-sex-specific rates according to the population distribution of England in 2014, to produce directly standardised rates for each year.

Patients typically have more than one injection procedure in a year. Variation in patient access to anti-VEGF injections within England might be evident in both the number of patients treated and the number of procedures performed. Therefore, we estimated indirectly standardised rates [16] per 100,000 population for both patients and procedures. We first calculated age-sex-specific rates for England in 2014/15, then multiplied these rates by the age-sex-specific population for the area of interest [17-19] (e.g. CCG) and summed the results. This produced the expected number of patients and procedures for that area, if it were to have the same age-sex-specific rates as England. The expected number was then compared to the observed number of patients and procedures for that area. A Poisson regression model was fitted to the observed counts, with the expected counts as an offset and socio-economic deprivation (using the overall score from the English Indices of Multiple Deprivation[20]) and ethnicity (% white British[21]) as predictive factors. The model was then used to predict new expected counts for each area based on deprivation and ethnicity, and form indirectly standardised patient and procedure ratios (observed / expected). Statistical analyses were conducted using Stata/MP 14.2 for Windows and we mapped variation in patient and procedure rates across England in 2014/15 using ArcGIS ArcMap 10.3.1 for Desktop.

EXPLORING VARIATION IN ACCESS IN THE SOUTH WEST REGION OF ENGLAND

For each CCG in the South West region of England we estimated the median age and gender proportion in order to explore any differences in the demographic profile of patients receiving anti-VEGF injections. We estimated the annual number of procedures per patient in 2014/15 to determine whether CCGs with high patient rates also had a higher number of procedures per patient. We also calculated and mapped indirectly standardised procedure ratios between Middle

For peer review only - http://bmjopen.bmj.com/site/about/guidelines.xhtml

Layer Super Output Areas (MSOA) to explore whether CCG boundaries or hospital catchment areas were more strongly associated with access to anti-VEGF injections.

ESTIMATING THE COST OF ANTI-VEGF INJECTIONS

We extracted data from NHS Digital Hospital prescribing costs reports [22] in order to estimate the costs of ranibizumab and aflibercept since 2008/9 when ranibizumab was first approved by NICE. The NHS Digital report summarises data collected from English NHS hospital pharmacies by the IMS Health Hospital Pharmacy Audit Index (HPAI). This includes medicines supplied to wards, outpatient clinics and satellite sites. Costs are calculated by IMS Health using standard price lists and therefore do not necessarily reflect any discounted prices paid by hospitals. Anti-VEGF therapy also incurs administration and monitoring costs; however, the IVAN trial indicated that, in the ranibizumab group, drug costs accounted for 80-88% of total costs [7]. Injection frequency and monitoring visits may be lower for aflibercept where fixed 2-monthly interval dosing is recommended after an initial period of monthly loading doses.

Due to the lack of drug coding in HES data it is impossible to accurately estimate the current use of bevacizumab for eye conditions. A study in 2015 based on 189 Freedom of Information requests across the UK estimated the relative prevalence of injections of ranibizumab, aflibercept and bevacizumab to be 61.1%, 36.0% and 2.8% respectively [23]. Subsequent legal challenges and the lack of Department of Health support for the use of bevacizumab have further increased the pressure on clinicians and commissioners not to use bevacizumab.

For peer review only - http://bmjopen.bmj.com/site/about/guidelines.xhtml

Results

THE USE AND COST OF ANTI-VEGF INTRAVITREAL INJECTIONS

In 2014/15 there were 388,031 intra-vitreal injection procedures, equivalent to 714 procedures per 100,000 population (Figure 2). This compares to 123,006 procedures in 2010/11 and represents a 215% increase in the number of procedures over the five-year period. Before 2007 intra-vitreal injections were rarely used. The rise in procedure rates since then is dramatic and there are no signs that it is levelling off.

Amongst episodes recorded in the HES APC dataset, the majority (62% in 2014/15) had diagnosis codes indicating nAMD (Figure 3). The relative use of diagnosis codes indicating DMO or RVO show small recent increases associated with NICE approvals for these indications in 2013. The estimated total cost of hospital prescribing of ranibizumab and aflibercept increased by 197% from £129 million to £383 million in the five years between 2010/11 and 2014/15 (Figure 4). The estimated total cost of ranibizumab and aflibercept had risen to £447 million in 2015/16, predominantly due to the increasing use of aflibercept.

VARIATION IN PATIENT ACCESS TO ANTI-VEGF THERAPY

In 2014/15 there was substantial variation in procedure rates between CCGs, even after adjusting for age, sex, deprivation, and ethnicity profiles (Figure 5a). The map demonstrates pockets of very high use (>150% times the expected rate), for example in the Bristol area and in parts of North London and Norfolk. There are also areas where procedure rates are less than 50% of the expected rate, for example in parts of Essex. There was less pronounced, but still substantial variation in patient rates between CCGs, after adjusting for age, sex, deprivation and ethnicity profiles (Figure 5b). Areas of the country with high procedure rates also tended to have higher proportions of their populations having treatment. In 2014/15 the ratio in patient rates between a 'high use' CCG at the 90th percentile and a 'low use' CCG at the 10th percentile was 4.98. This ratio has only slightly decreased

For peer review only

since 2010/11, when the ratio was 6.07, indicating that equality of access to care is only slowly improving over time.

Examining the South West region of England in more detail (Figures 6a and 6b), it is evident that there is substantial variation in access to anti-VEGF injections within CCGs. For example, in both Somerset and Wiltshire there are areas of relatively high procedure rates closer to the major conurbations of Bath, Bristol and Swindon. Procedure rates are much lower than expected in other areas of these CCGs, even though these other areas are served by local hospitals. The age and gender profile of patients receiving anti-VEGF therapy was similar between CCGs (Table 2). Median age ranged from 77 to 82 years and between 39% and 49% of patients were male. There was twofold variation in the number of procedures per patient per year ranging from 2.9 in Cornwall to 5.9 in South Gloucestershire. The standardised CCG patient rate was strongly correlated with the number of procedures per patient (Spearman's Rho = 0.71, $p < .001$).

For peer review only - http://bmjopen.bmj.com/site/about/guidelines.xhtml

Discussion

STATEMENT OF PRINCIPAL FINDINGS

There is no evidence that the dramatic growth in the use of high cost anti-VEGF injections in ophthalmology is slowing down. Injection procedures increased by 215% between 2010/11 and 2014/15. There are large inequalities in access to anti-VEGF therapy. This inequality is not explained by differences in demographics and occurs both within and between CCGs. Procurement of high cost anti-VEGF drugs has placed a large and increasing strain on the NHS. In 2015/16, the NHS spent an estimated £447 million on drug costs alone, equivalent to the entire annual budget of a large CCG.

STRENGTHS AND WEAKNESSES OF THE STUDY

Our study summarises new data on nationwide access to effective, but high cost ophthalmology drugs. Using population denominators allows us to standardise our analysis for factors, such as age and socio-economic deprivation, which will affect the need for care. HES data records patients' area of residence, so we compare access based on place of residence rather than place of treatment. The combination of HES APC and OP datasets is essential in order to accurately monitor intravitreal injections over time. As reimbursement payments depend on procedure coding, there is an incentive for providers to fully code procedures. However, the lack of a specific anti-VEGF drug code in HES is a limitation. Other similar procedures such as the intravitreal injection of dexamethasone implants have distinct OPCS procedure codes, although we cannot exclude the possibility of miscoding. We observed similar temporal trends in two independent data sources measuring procedures (i.e. HES) and drug costs (i.e. HPAI) which provides reassurance about the validity of our time-trend analyses. Previous work has demonstrated that HES generally has good sensitivity in identifying new procedures, but may lack specificity particularly in the absence of a single specific procedure code [24]. The confidential nature of drug discounts mean that our estimate of drug costs

For peer review only - http://bmjopen.bmj.com/site/about/guidelines.xhtml

will be too high if CCGs have negotiated substantial discounts. Conversely, the exclusion of administration and monitoring costs make our estimates conservative and there is a substantial economic burden for patients. Travel and post-appointment recovery time have a large impact on patients and carers [25].

COMPARISON WITH OTHER STUDIES

Between 1989/90 and 2008/9, Keenan et al observed an increase in intravitreal injection rates from 0.4 per 100,000 to 59.5 per 100.000 with wide variations in access between different local authorities in England [26]. Our data demonstrate that this rapid growth has continued and even accelerated in the following six years. We were also able to explore variation in access to care at a more granular level and demonstrate that access varies markedly both within and between CCGs, suggesting that variations may be due to the availability of trained staff and facilities in local hospitals rather than CCG-wide policies. Using insurance data, Parikh et al observed a continually increasing trend in anti-VEGF use in the US between 2006 and 2015 [27]. In line with our study, Parikh et al also found that anti-VEGF drug use was predominantly for nAMD. It is impossible to directly compare England and US rates as the denominator used in the Parikh et al study excludes patients without commercial or Medicare Advantage insurance. However, anti-VEGF therapy rates in the US and UK appear to be of the same order of magnitude. Specific coding of anti-VEGF drugs allowed Parikh et al to document the rise in the use of aflibercept (28% of all anti-VEGF injections in 2015) and the concurrent levelling off in the use of ranibizumab (17%) and bevacizumab (55%). This is in stark contrast to England where ranibizumab and aflibercept predominate.

IMPLICATIONS FOR POLICYMAKERS AND CLINICIANS

Based on the IVAN trial, Dakin et al estimated that the NHS could save £102 million per year by switching from ranibizumab to bevacizumab [7]. In the US, Hutton et al estimated that a similar switch would save Medicare $18 billion over a 10-year period [28]. Given the lack of political and

For peer review only - http://bmjopen.bmj.com/site/about/guidelines.xhtml

regulatory support for clinicians to use bevacizumab, it is unsurprising that most in England do not use it. This has led to the very unusual situation whereby the NHS is paying for more expensive therapy than healthcare insurers in the US. The patent on ranibizumab is due to expire in Europe in 2022 [29]; in the meantime, based on current trends, the NHS may spend billions on anti-VEGF injections, before biosimilar drugs become available. Our data, demonstrating large variations between CCGs in the numbers of patients accessing anti-VEGF services also suggests that there is considerable potential unmet need in some areas of the country. Given that many CCGs are already in financial deficit, most will struggle to afford to treat more patients unless they are able to switch to bevacizumab. The political decision not to support NHS use of bevacizumab in eye conditions is in stark contrast to decisions taken in other EU countries and has very large negative consequences for NHS patients.

In April 2017, NICE introduced a budget impact test for new technologies under appraisal that are expected to cost more than £20 million per annum. This allows NHS England up to three years to conduct commercial negotiations on price rather than the current requirement to fund within 90 days [30]. This policy should strengthen the future negotiating power of the NHS for expensive drugs. However, it does not address the fundamental problem that NICE has not included cost-effective but unlicensed drugs in technology appraisals or clinical guideline recommendations.

The widespread variation in procedure rates that we observed might be due to travel distance affecting patient access to diagnostic and therapeutic services, particularly in more remote areas. Lengthy travel distance is associated treatment discontinuance [31]. However, many of the low injection rate areas in the South West of England are close to hospitals. Other unmeasured factors, such as the importance of vision for work or usual activities, may also be associated with patient uptake and compliance with anti VEGF therapy. The frequency of injections per patient per year was lower than reported in trials of therapy initiation. The averages in our study will be drawn

For peer review only - http://bmjopen.bmj.com/site/about/guidelines.xhtml

downwards due to patients starting and discontinuing therapy partway through the year and by patients who have been on anti-VEGF therapy for many years and need fewer injections.

UNANSWERED QUESTIONS AND FUTURE RESEARCH

Given the high cost of the drugs, it is imperative that clinical services are organised to deliver care efficiently. For example, nurse-administered injections [32], treat and extend protocols [33], optometrist-led monitoring [34] and stereotactic radiotherapy [35] all offer the potential to reduce costs and require stronger evidence. The wide variation between CCGs in the number of injections per patient represents a natural experiment. Observational studies could compare costs and outcomes between areas of the country with high and low injection frequency. However, the lack of a nationwide register of patients receiving anti-VEGF injections limits the potential to conduct this research.

CONCLUSIONS

Anti-VEGF therapy is effective in most cases and has improved the quality of life of many thousands of patients in England. However, the spiralling cost of anti-VEGF therapy affects the ability of the NHS to provide care for other patients. NICE was established in 1999 to ensure that patients in England have equal access to cost-effective care. In this instance, it has not been able to achieve either goal.

For peer review only - http://bmjopen.bmj.com/site/about/guidelines.xhtml

Acknowledgements:

Hospital Episode Statistics were provided by NHS Digital under data sharing agreement (NIC-17875-X7K1V) with the University of Bristol. Copyright © 2016, re-used with the permission of The Health & Social Care Information Centre. All rights reserved.

Funding:

William Hollingworth and Tim Jones receive funding from the National Institute for Health Research Collaboration for Leadership in Applied Health Research and Care (NIHR CLAHRC) West. The views expressed are those of the authors and not necessarily those of the NHS, the NIHR or the Department of Health. The funder had no role in the study design, collection, analysis and interpretation of data or in the writing of the report and the decision to submit the article for publication.

Competing Interests:

All authors have completed the Unified Competing Interest form (available on request from the corresponding author) and declare: no support from any organisation for the submitted work; no financial relationships with any organisations that might have an interest in the submitted work in the previous three years, no other relationships or activities that could appear to have influenced the submitted work.

Copyright statement:

The Corresponding Author has the right to grant on behalf of all authors and does grant on behalf of all authors, a worldwide licence to the Publishers and its licensees in perpetuity, in all forms, formats and media (whether known now or created in the future), to i) publish, reproduce, distribute, display and store the Contribution, ii) translate the Contribution into other languages, create adaptations, reprints, include within collections and create summaries, extracts and/or, abstracts of the Contribution, iii) create any other derivative work(s) based on the Contribution, iv) to exploit all subsidiary rights in the Contribution, v) the inclusion of electronic links from the Contribution to third party material where-ever it may be located; and, vi) licence any third party to do any or all of the above.

We attest that we have obtained appropriate permissions and paid any required fees for use of copyright protected materials.

Transparency declaration:

The lead author affirms that the manuscript is an honest, accurate, and transparent account of the study being reported; that no important aspects of the study have been omitted; and that any discrepancies from the study as planned (and, if relevant, registered) have been explained.

Details of contributors:

WH initiated and designed the study, contributed to the data analysis and interpretation of data and drafted and revised the paper. He is guarantor. TJ contributed to the study design, cleaned and analysed the data, contributed to the interpretation of data and revised the draft paper. BR and TP contributed to the interpretation of data and revised the draft paper. All authors approved the final version of the paper. All authors had full access to all of the data in the study and can take responsibility for the integrity of the data and the accuracy of the data analysis.

Ethical approval:

For peer review only

The paper describes work based on anonymised individual episode records provided under a data sharing agreement between NHS Digital and the University of Bristol. The data were not linked to other datasets or re-identified. Ethical approval was not required.

Data sharing:
No additional data available.

For peer review only

For peer review only - http://bmjopen.bmj.com/site/about/guidelines.xhtml

Figure 1 – OPCS codes used to identify anti-VEGF injections: 2014/15

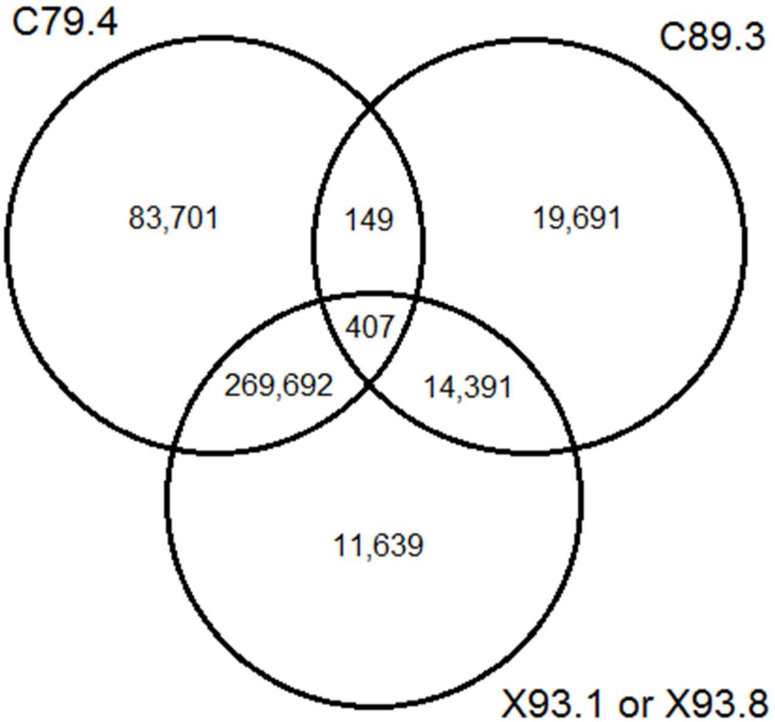

Note: 2,479 eye injection procedures were excluded due to combination with X93.2 or X93.3 high-cost drug codes.

For peer review only - http://bmjopen.bmj.com/site/about/guidelines.xhtml

Figure 2. Directly-standardised intravitreal injection procedure rates, England, 2005/06-2014/15

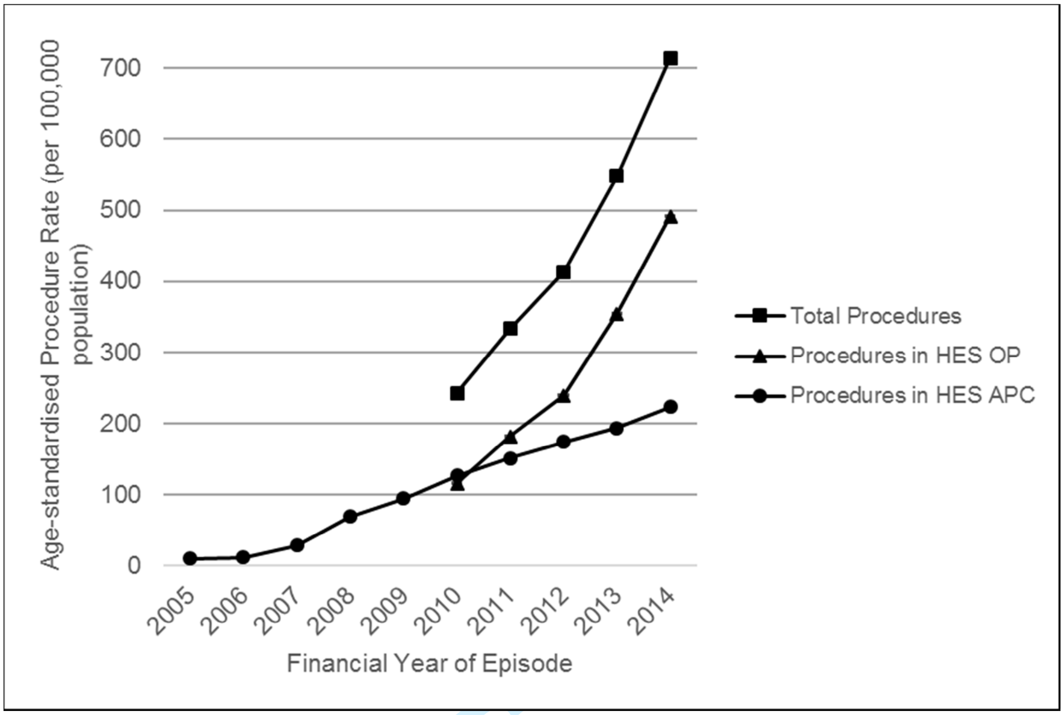

Note: OP = Outpatient; APC = Admitted Patient Care

For peer review only - http://bmjopen.bmj.com/site/about/guidelines.xhtml

Figure 3. Percentage of total eye injection procedures in HES APC for different primary diagnoses, England, 2008/09-2014/15.

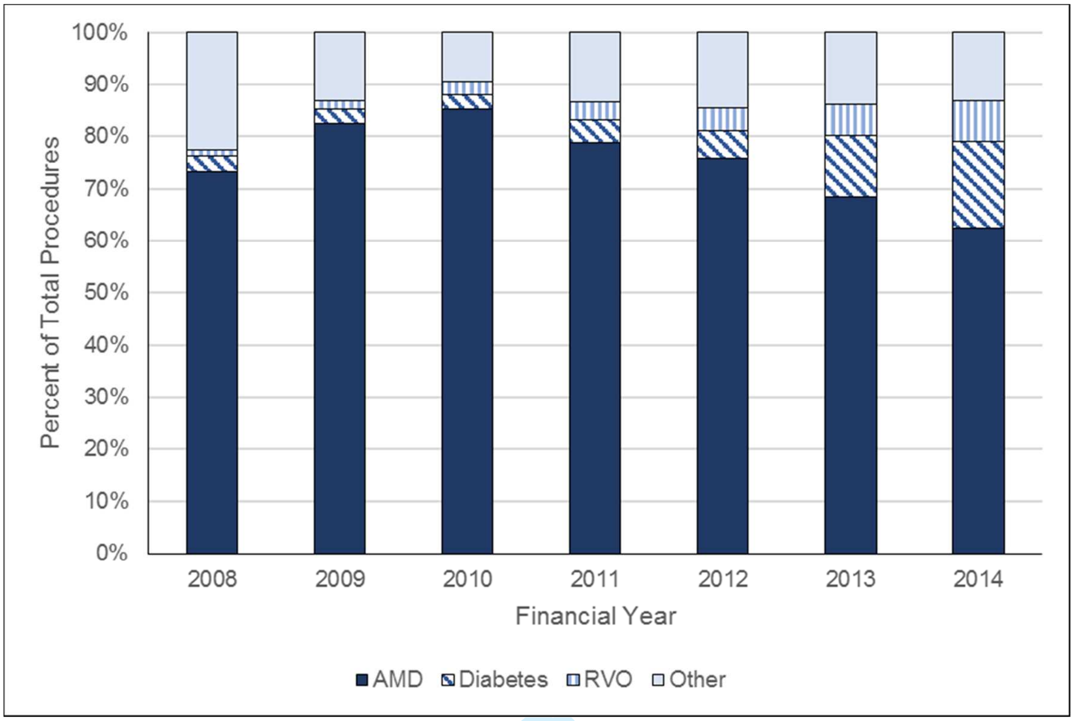

Note: nAMD = Neovascular Age-Related Macular Degeneration; DMO = Diabetic Macular Oedema; RVO = Retinal Vein Occlusion; Due to very small numbers of procedures for Myopic Choroidal Neo-Vascularisation (<=10 per year), this was included in 'Other'.

review only

For peer review only - http://bmjopen.bmj.com/site/about/guidelines.xhtml

Figure 4. Hospital prescribing costs for Ranibizumab and Aflibercept, 2008/09 to 2015/16

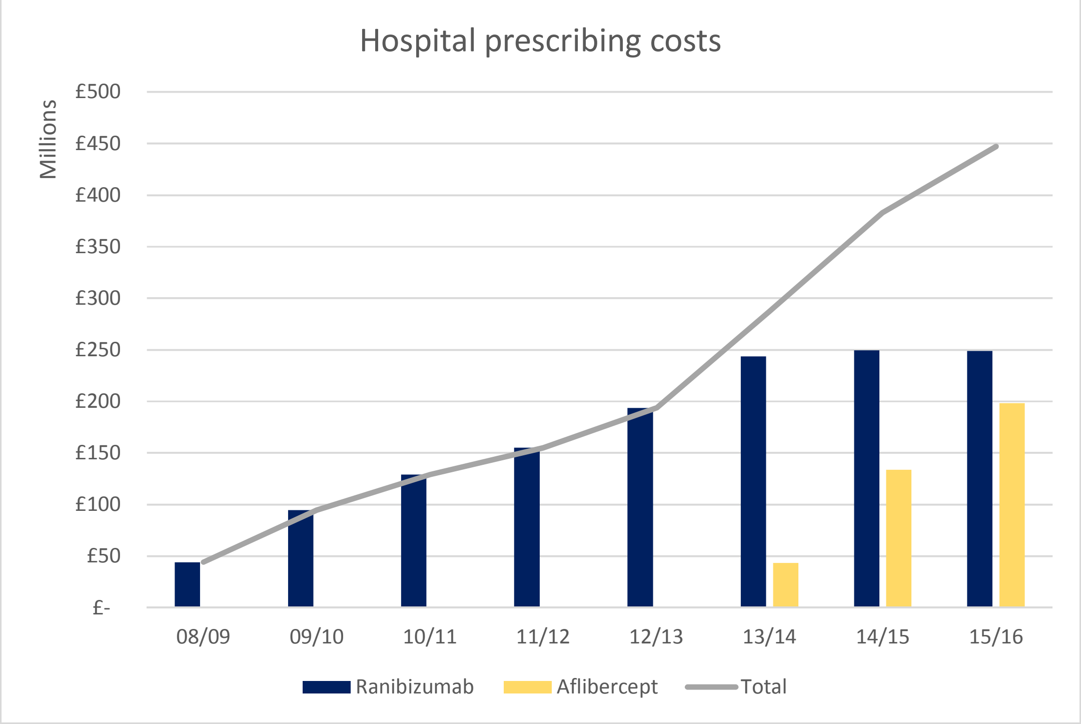

For peer review only - http://bmjopen.bmj.com/site/about/guidelines.xhtml

Figure 5a. Indirectly age-sex-deprivation-ethnicity standardised procedure ratios for combined eye injection procedures (OPCS-4: C794 and C893) by CCG in England, 2014/15.

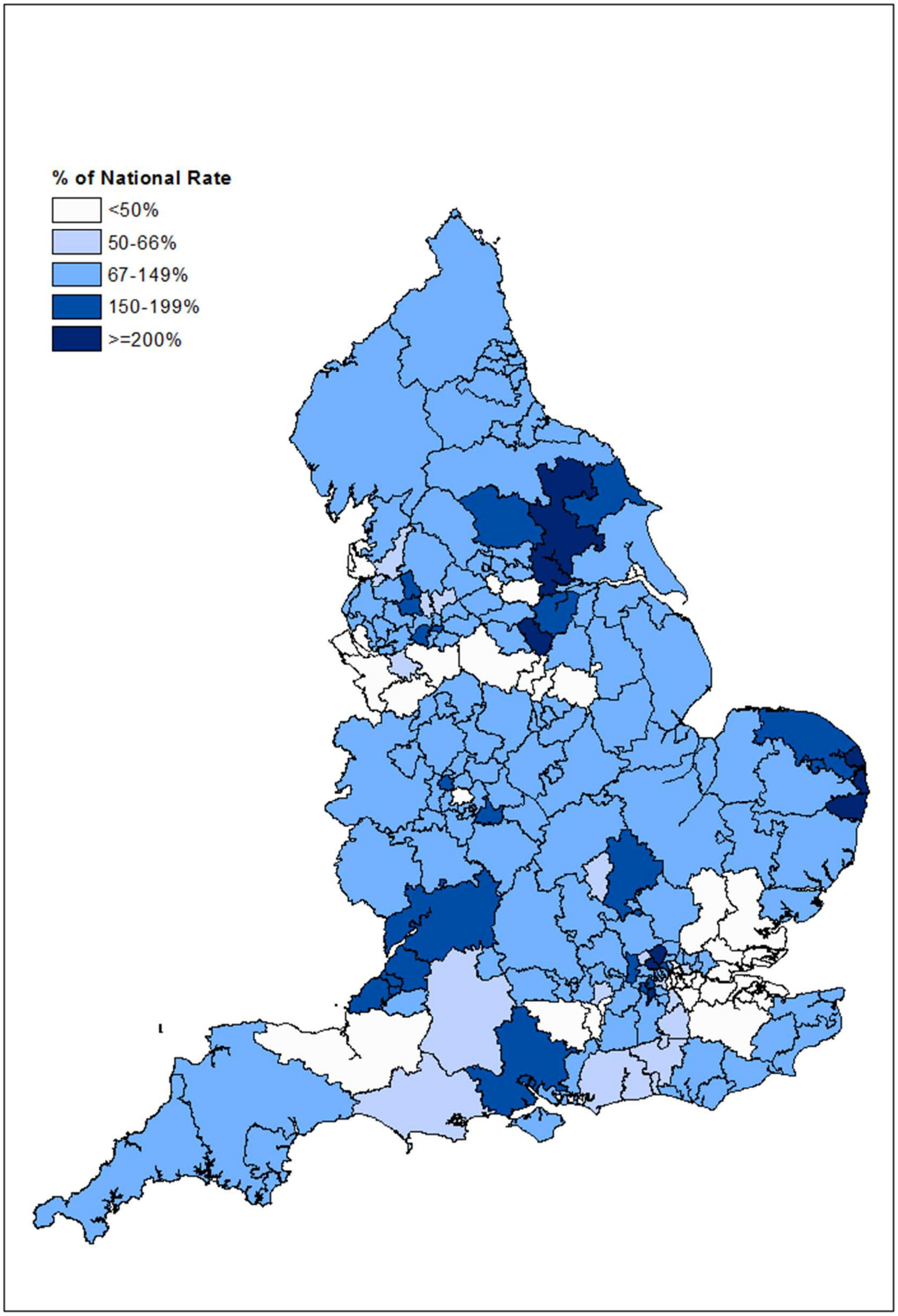

For peer review only - http://bmjopen.bmj.com/site/about/guidelines.xhtml

Figure 5b. Indirectly age-sex-deprivation-ethnicity standardised patient ratios for combined eye injection procedures (OPCS-4: C794 and C893) by CCG in England, 2014/15.

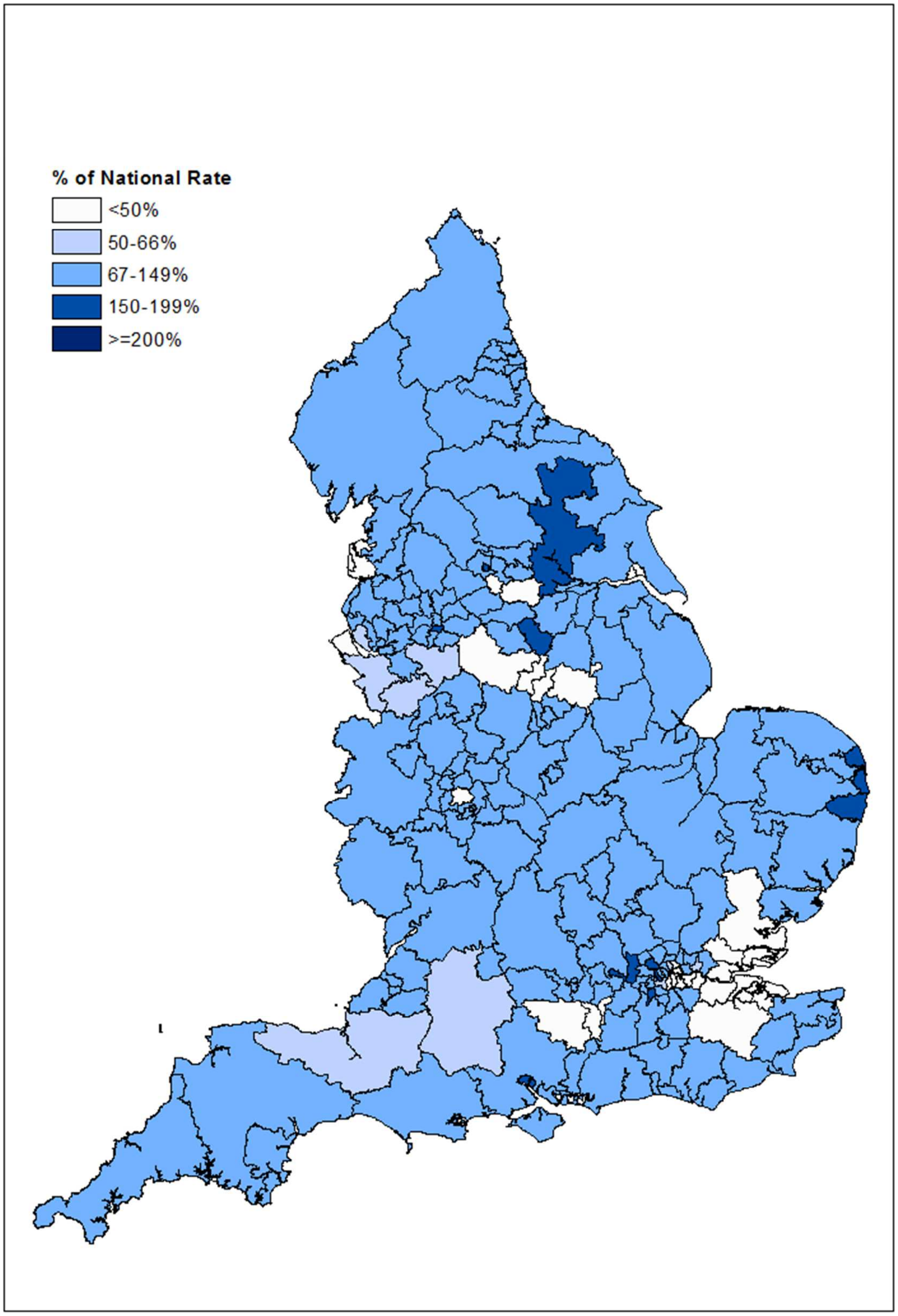

Figure 6a. Indirectly standardised procedure ratios for combined eye injection procedures by MSOA in SW England, 2014/15

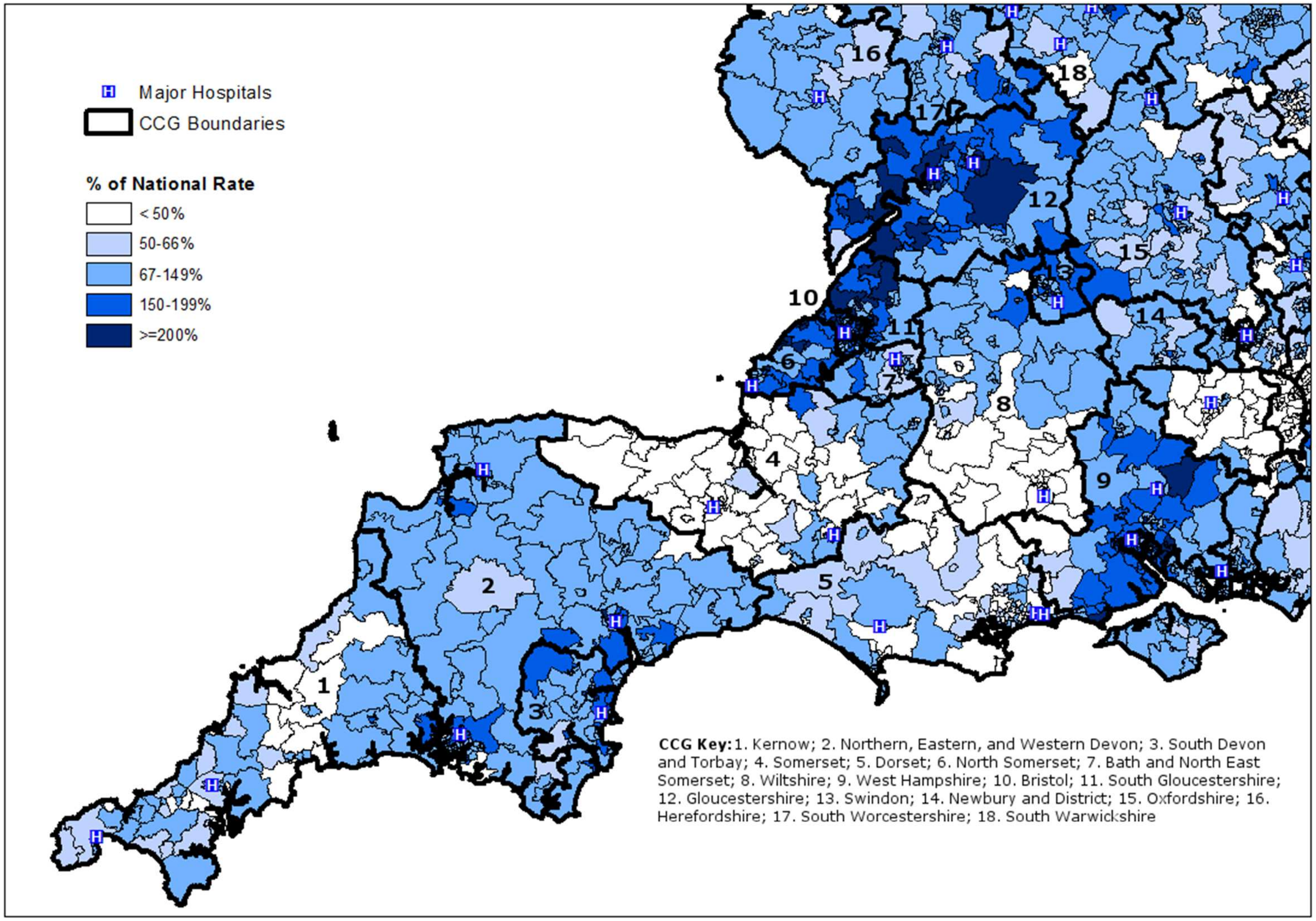

Note: Major hospitals marked including those in NHS Trusts providing 100+ procedures.

For peer review only - http://bmjopen.bmj.com/site/about/guidelines.xhtml

Figure 6b. Indirectly standardised patient ratios for combined eye injection procedures by MSOA in SW England, 2014/15

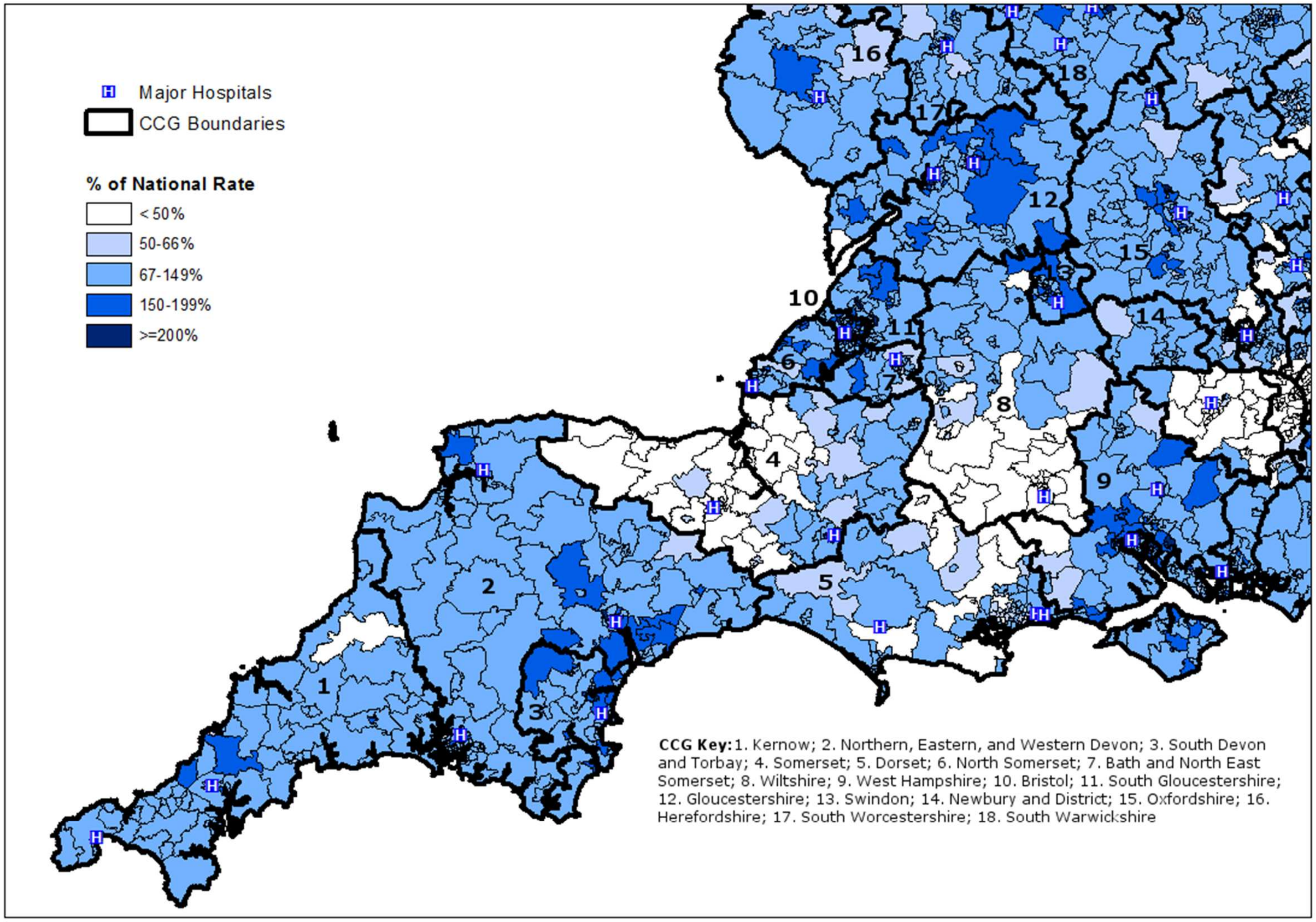

Note: Major hospitals marked including those in NHS Trusts providing 100+ procedures.

For peer review only - http://bmjopen.bmj.com/site/about/guidelines.xhtml

Table 1 – NICE appraisals for anti-VEGF therapies

Condition	Ranibizumab	Aflibercept
nAMD	Recommend 8/2008 [TA 155]	Recommend 7/2013 [TA 294]
DMO	Reject 11/2011 [TA 237]<br>Recommend 2/2013 [TA 274]	Recommend 7/2015 [TA 346]
RVO	Recommend 5/2013 [TA 294]	Recommend 2/2014 [TA 305]
MCNV	Recommend 11/2013 [TA 298]	Recommend 9/2016 [TA 409]

For peer review only

Table 2. Demographics of anti-VEGF procedures in South West CCGs, 2014/15; ordered by procedures per patient.

CCG Name	% Male	Median Age	Procedures per Patient (95% CI)	Procedure Rate* (95% CI**)	Patient Rate* (95% CI)
NHS Kernow CCG	43	79	2.86 (2.75-2.97)	522 (490-556)	172 (191-104)
NHS Dorset CCG	43	82	3.32 (3.21-3.43)	419 (394-445)	122 (136-74)
NHS Herefordshire CCG	38	80	3.34 (3.14-3.53)	631 (569-700)	175 (210-110)
NHS Somerset CCG	45	78	3.48 (3.27-3.68)	304 (276-334)	92 (106-57)
NHS South Warwickshire CCG	44	79	3.63 (3.46-3.8)	773 (708-844)	206 (240-128)
NHS Oxfordshire CCG	40	81	3.63 (3.5-3.76)	723 (679-771)	197 (220-120)
NHS Northern Eastern and Western Devon CCG	41	81	4.05 (3.95-4.16)	830 (792-869)	201 (218-121)
NHS Newbury and District CCG	41	79	4.14 (3.72-4.55)	637 (530-764)	136 (186-92)
NHS Wiltshire CCG	46	79	4.17 (3.95-4.39)	459 (419-502)	103 (121-64)
NHS South Devon and Torbay CCG	40	81	4.2 (4.05-4.35)	923 (860-991)	206 (235-127)
NHS South Worcestershire CCG	42	79	4.36 (4.16-4.56)	875 (806-950)	188 (217-116)
NHS Bath and North East Somerset CCG	49	81	4.44 (4.14-4.73)	735 (650-831)	152 (189-97)
NHS Swindon CCG	44	77	4.54 (4.26-4.82)	1034 (928-1153)	216 (260-137)
NHS West Hampshire CCG	42	80	5 (4.85-5.15)	1125 (1063-1190)	221 (244-133)
NHS North Somerset CCG	43	80	5.43 (5.15-5.71)	1236 (1130-1352)	217 (253-135)
NHS Bristol CCG	43	80	5.49 (5.26-5.72)	1346 (1248-1451)	237 (270-146)
NHS Gloucestershire CCG	42	81	5.51 (5.33-5.68)	1263 (1194-1336)	226 (249-137)
NHS South Gloucestershire CCG	46	79	5.93 (5.65-6.22)	1379 (1264-1505)	222 (258-138)

** Procedure rate and patient rate are the indirectly standardised rates per 100,000 population*

*** 95% CIs on the procedure rate are calculated using Stukel et al.'s method for recurrent outcomes[36]*

For peer review only - http://bmjopen.bmj.com/site/about/guidelines.xhtml

References

1. Steinbrook, R., *The price of sight--ranibizumab, bevacizumab, and the treatment of macular degeneration.* N Engl J Med, 2006. **355**(14): p. 1409-12.
2. National Institute for Health and Care Excellence, *Ranibizumab and pegaptanib for the treatment of age-related macular degeneration*. 2008.
3. National Institute for Health and Care Excellence, *Ranibizumab for treating diabetic macular oedema*. 2013.
4. BMJ Group and Pharmaceutical Press, *British National Formulary*. 2017: London.
5. Poku, E., et al., *Bevacizumab in eye conditions: Issues related to quality, use, efficiency and safety*. 2012, NICE Decision Support Unit: Sheffield.
6. Solomon, S.D., et al., *Intravitreal Bevacizumab Versus Ranibizumab for Treatment of Neovascular Age-Related Macular Degeneration: Findings from a Cochrane Systematic Review.* Ophthalmology, 2016. **123**(1): p. 70-77 e1.
7. Dakin, H.A., et al., *Cost-effectiveness of ranibizumab and bevacizumab for age-related macular degeneration: 2-year findings from the IVAN randomised trial.* BMJ Open, 2014. **4**(7): p. e005094.
8. Maguire, M., et al., *Serious Adverse Events with Bevacizumab or Ranibizumab for Age-Related Macular Degeneration: Meta-analysis of Individual Patient Data.* Ophthalmology Retina, In Press.
9. Ross, E.L., et al., *Cost-effectiveness of Aflibercept, Bevacizumab, and Ranibizumab for Diabetic Macular Edema Treatment: Analysis From the Diabetic Retinopathy Clinical Research Network Comparative Effectiveness Trial.* JAMA Ophthalmol, 2016. **134**(8): p. 888-96.
10. Scott, I.U., et al., *Effect of Bevacizumab vs Aflibercept on Visual Acuity Among Patients With Macular Edema Due to Central Retinal Vein Occlusion: The SCORE2 Randomized Clinical Trial.* JAMA, 2017. **317**(20): p. 2072-2087.
11. Lotery, A. and C. MacEwen, *What is stopping the NHS from using bevacizumab for macular degeneration and other retinal disorders?* BMJ, 2014. **349**: p. g6887.
12. Freeman, G., *Letter to NHS Southern Derbyshire CCG*, Department of Health, Editor. 2015.
13. Formoso, G., et al., *A clearer view of evidence in treating macular degeneration: off-label policies and independent research.* Cochrane Database Syst Rev, 2014(9): p. ED000090.
14. National Institute for Health and Care Excellence, *Guideline scope age-related macular degeneration: diagnosis and management* 2015, NICE.
15. Health and Social Care Information Centre, *Hospital Episode Statistics (HES) Analysis Guide*. 2015.
16. Anon, *Standardization: a classic epidemiological method for the comparison of rates.* Epidemiological Bulletin, 2002. **23**(3): p. 9-12.
17. Office for National Statistics. *Lower Super Output Area Mid-Year Population Estimates* [cited 2016; Available from: https://www.ons.gov.uk/peoplepopulationandcommunity/populationandmigration/populationestimates/datasets/lowersuperoutputareamidyearpopulationestimates.
18. Office for National Statistics. *Lower Layer Super Output Area (2011) to Clinical Commissioning Group to Local Authority District (July 2015) Lookup in England*. [cited 2016; Available from: http://ons.maps.arcgis.com/home/item.html?id=7de88cd2369d4006925de49e8f3f2e27.
19. Office for National Statistics. *Output Area to Lower Layer Super Output Area to Middle Layer Super Output Area to Local Authority District (December 2011) Lookup in England and Wales*. [cited 2016; Available from: http://ons.maps.arcgis.com/home/item.html?id=baf6566ca08949c6bbd61ff81d9526da.

For peer review only - http://bmjopen.bmj.com/site/about/guidelines.xhtml

20. Government, D.o.C.a.L. *English Indices of Deprivation 2015*. [cited 2016; Available from: https://www.gov.uk/government/statistics/english-indices-of-deprivation-2015.
21. Nomis; Office for National Statistics. *Ethnic group of the usual resident population of England and Wales as at census day, 27 March 2011*. [cited 2016; Available from: https://www.nomisweb.co.uk/census/2011/ks201ew.
22. NHS Digital, *Prescribing Costs in Hospitals and the Community*. 2016.
23. Di Simplicio, S., et al. *A UK survey of anti-VEGF use for the eye in January 2015*. in *The Association for Research in Vision and Ophthalmology Annual Meeting*. 2017. Baltimore, MD.
24. Patrick, H., et al., *Monitoring the use and outcomes of new devices and procedures: how does coding affect what Hospital Episode Statistics contribute? Lessons from 12 emerging procedures 2006-10.* J Public Health (Oxf), 2013. **35**(1): p. 132-8.
25. Prenner, J.L., et al., *Disease Burden in the Treatment of Age-Related Macular Degeneration: Findings From a Time-and-Motion Study.* Am J Ophthalmol, 2015. **160**(4): p. 725-31 e1.
26. Keenan, T.D., C.J. Wotton, and M.J. Goldacre, *Trends over time and geographical variation in rates of intravitreal injections in England.* Br J Ophthalmol, 2012. **96**(3): p. 413-8.
27. Parikh, R., et al., *Trends of Anti-Vascular Endothelial Growth Factor Use in Ophthalmology Among Privately Insured and Medicare Advantage Patients.* Ophthalmology, 2016.
28. Hutton, D., et al., *Switching to less expensive blindness drug could save medicare part B $18 billion over a ten-year period.* Health Aff (Millwood), 2014. **33**(6): p. 931-9.
29. Generics and biosimilars initiative. *Biosimilars of ranibizumab*. 2015 18 May 2017]; Available from: http://gabionline.net/Biosimilars/General/Biosimilars-of-ranibizumab.
30. Charlton, V., et al., *Cost effective but unaffordable: an emerging challenge for health systems.* BMJ, 2017. **356**: p. j1402.
31. Boulanger-Scemama, E., et al., *Ranibizumab for exudative age-related macular degeneration: A five year study of adherence to follow-up in a real-life setting.* J Fr Ophtalmol, 2015. **38**(7): p. 620-7.
32. Austeng, D., et al., *Nurse-administered intravitreal injections of anti-VEGF: study protocol for noninferiority randomized controlled trial of safety, cost and patient satisfaction.* BMC Ophthalmol, 2016. **16**(1): p. 169.
33. Chin-Yee, D., et al., *A systematic review of as needed versus treat and extend ranibizumab or bevacizumab treatment regimens for neovascular age-related macular degeneration.* Br J Ophthalmol, 2016. **100**(7): p. 914-917.
34. Violato, M., et al., *Cost-effectiveness of community versus hospital eye service follow-up for patients with quiescent treated age-related macular degeneration alongside the ECHoES randomised trial.* BMJ Open, 2016. **6**(10): p. e011121.
35. Neffendorf, J.E., et al., *StereoTactic radiotherapy for wet Age-Related macular degeneration (STAR): study protocol for a randomised controlled clinical trial.* Trials, 2016. **17**(1): p. 560.
36. Stukel, T.A., et al., *Standardized Rates of Recurrent Outcomes.* Statistics in Medicine, 1994. **13**(17): p. 1781-1791.

For peer review only

For peer review only - http://bmjopen.bmj.com/site/about/guidelines.xhtml

The RECORD statement – checklist of items, extended from the STROBE statement, that should be reported in observational studies using routinely collected health data.

	Item No.	STROBE items	Location in manuscript where items are reported	RECORD items	Location in manuscript where items are reported
**Title and abstract**					
	1	(a) Indicate the study's design with a commonly used term in the title or the abstract (b) Provide in the abstract an informative and balanced summary of what was done and what was found	P2, L6<br><br>P2, L6-27	RECORD 1.1: The type of data used should be specified in the title or abstract. When possible, the name of the databases used should be included.<br><br>RECORD 1.2: If applicable, the geographic region and timeframe within which the study took place should be reported in the title or abstract.<br><br>RECORD 1.3: If linkage between databases was conducted for the study, this should be clearly stated in the title or abstract.	P2, L6 & 7<br><br>P2, L6-10<br><br>N/A
**Introduction**					
Background rationale	2	Explain the scientific background and rationale for the investigation being reported	P4-5		
Objectives	3	State specific objectives, including any prespecified hypotheses	P5, L21-24		
**Methods**					
Study Design	4	Present key elements of study design early in the paper	P6-9		
Setting	5	Describe the setting, locations, and relevant dates, including periods of recruitment, exposure, follow-up, and data collection	P6, L2-24<br>P9, L4-13		
Participants	6	*(a) Cohort study* - Give the eligibility criteria, and the	P6, L12-24<br>P7, L1-18	RECORD 6.1: The methods of study population selection (such as codes or	P6, L12-24<br>P7, L1-18

For peer review only

		sources and methods of selection of participants. Describe methods of follow-up<br>*Case-control study* - Give the eligibility criteria, and the sources and methods of case ascertainment and control selection. Give the rationale for the choice of cases and controls<br>*Cross-sectional study* - Give the eligibility criteria, and the sources and methods of selection of participants<br><br>*(b) Cohort study* - For matched studies, give matching criteria and number of exposed and unexposed<br>*Case-control study* - For matched studies, give matching criteria and the number of controls per case	N/A	algorithms used to identify subjects) should be listed in detail. If this is not possible, an explanation should be provided.<br><br>RECORD 6.2: Any validation studies of the codes or algorithms used to select the population should be referenced. If validation was conducted for this study and not published elsewhere, detailed methods and results should be provided.<br><br>RECORD 6.3: If the study involved linkage of databases, consider use of a flow diagram or other graphical display to demonstrate the data linkage process, including the number of individuals with linked data at each stage.	P6, L12-17<br><br>N/A
Variables	7	Clearly define all outcomes, exposures, predictors, potential confounders, and effect modifiers. Give diagnostic criteria, if applicable.	P7, L20-24<br>P8, L1-18	RECORD 7.1: A complete list of codes and algorithms used to classify exposures, outcomes, confounders, and effect modifiers should be provided. If these cannot be reported, an explanation should be provided.	P7, L20-24<br>P8, L1-18
Data sources/ measurement	8	For each variable of interest, give sources of data and details of methods of assessment (measurement).<br>Describe comparability of assessment methods if there is more than one group	P7 – P9		
Bias	9	Describe any efforts to address potential sources of bias	P7, L20-24<br>P8, L1-18		
Study size	10	Explain how the study size was	N/A – there was no		

For peer review only

		arrived at	sample size calculation		
Quantitative variables	11	Explain how quantitative variables were handled in the analyses. If applicable, describe which groupings were chosen, and why	P7, L20-24<br>P8, L1-18		
Statistical methods	12	(a) Describe all statistical methods, including those used to control for confounding<br>(b) Describe any methods used to examine subgroups and interactions<br>(c) Explain how missing data were addressed<br>(d) *Cohort study* - If applicable, explain how loss to follow-up was addressed<br>*Case-control study* - If applicable, explain how matching of cases and controls was addressed<br>*Cross-sectional study* - If applicable, describe analytical methods taking account of sampling strategy<br>(e) Describe any sensitivity analyses	P7, L20-24<br>P8, L1-18<br><br>N/A<br><br>N/A<br><br>N/A		
Data access and cleaning methods		..		RECORD 12.1: Authors should describe the extent to which the investigators had access to the database population used to create the study population.<br><br>RECORD 12.2: Authors should provide information on the data cleaning methods used in the study.	P6, L1-11

For peer review only

				P7, L20-24<br>P8, L1-18	
Linkage		..		RECORD 12.3: State whether the study included person-level, institutional-level, or other data linkage across two or more databases. The methods of linkage and methods of linkage quality evaluation should be provided.	N/A
**Results**					
Participants	13	(a) Report the numbers of individuals at each stage of the study (*e.g.*, numbers potentially eligible, examined for eligibility, confirmed eligible, included in the study, completing follow-up, and analysed)<br>(b) Give reasons for non-participation at each stage.<br>(c) Consider use of a flow diagram	Figure 1, P10, L1-7	RECORD 13.1: Describe in detail the selection of the persons included in the study (*i.e.,* study population selection) including filtering based on data quality, data availability and linkage. The selection of included persons can be described in the text and/or by means of the study flow diagram.	Figure 1
Descriptive data	14	(a) Give characteristics of study participants (*e.g.*, demographic, clinical, social) and information on exposures and potential confounders<br>(b) Indicate the number of participants with missing data for each variable of interest<br>(c) *Cohort study* - summarise follow-up time (*e.g.*, average and total amount)	Table 2<br><br>N/A		
Outcome data	15	*Cohort study* - Report numbers of outcome events or summary measures over time<br>*Case-control study* - Report numbers in each exposure category, or summary measures of exposure	Figures 2-6		

For peer review only

		Cross-sectional study - Report numbers of outcome events or summary measures			
Main results	16	(a) Give unadjusted estimates and, if applicable, confounder-adjusted estimates and their precision (e.g., 95% confidence interval). Make clear which confounders were adjusted for and why they were included<br>(b) Report category boundaries when continuous variables were categorized<br>(c) If relevant, consider translating estimates of relative risk into absolute risk for a meaningful time period	Table 2<br>N/A<br>N/A		
Other analyses	17	Report other analyses done—e.g., analyses of subgroups and interactions, and sensitivity analyses	N/A		
**Discussion**					
Key results	18	Summarise key results with reference to study objectives	P12, L1-9		
Limitations	19	Discuss limitations of the study, taking into account sources of potential bias or imprecision. Discuss both direction and magnitude of any potential bias	P12 L11-24<br>P13, L1-4	RECORD 19.1: Discuss the implications of using data that were not created or collected to answer the specific research question(s). Include discussion of misclassification bias, unmeasured confounding, missing data, and changing eligibility over time, as they pertain to the study being reported.	P12 L11-24<br>P13, L1-4
Interpretation	20	Give a cautious overall interpretation of results considering objectives, limitations, multiplicity of analyses, results from similar studies, and other relevant	P15, L13-17		

For peer review only

		evidence			
Generalisability	21	Discuss the generalisability (external validity) of the study results	P13, L6-20		
**Other Information**					
Funding	22	Give the source of funding and the role of the funders for the present study and, if applicable, for the original study on which the present article is based	P16		
Accessibility of protocol, raw data, and programming code		..		RECORD 22.1: Authors should provide information on how to access any supplemental information such as the study protocol, raw data, or programming code.	N/A

*Reference: Benchimol EI, Smeeth L, Guttmann A, Harron K, Moher D, Petersen I, Sørensen HT, von Elm E, Langan SM, the RECORD Working Committee. The REporting of studies Conducted using Observational Routinely-collected health Data (RECORD) Statement. *PLoS Medicine* 2015; in press.

*Checklist is protected under Creative Commons Attribution (CC BY) license.

For peer review only

Changes in the frequency and cost of anti-vascular endothelial therapy, and inequalities in access, in England between 2005 and 2015

Journal:	*BMJ Open*
Manuscript ID	bmjopen-2017-018289.R1
Article Type:	Research
Date Submitted by the Author:	16-Aug-2017

Complete List of Authors:	Hollingworth, William; University of Bristol, School of Social and Community Medicine<br>Jones, Tim; NIHR CLAHRC West,<br>Reeves, Barnaby; University of Bristol, Bristol Heart Institute<br>Peto, Tunde; Queen's University Belfast Faculty of Medicine Health and Life Sciences

**Primary Subject Heading**:	Health policy
Secondary Subject Heading:	Health economics, Ophthalmology, Evidence based practice, Public health
Keywords:	Vascular Endothelial Growth Factor A, Drug Costs, macular degeneration, macular edema, England, Geography

SCHOLARONE™ Manuscripts

Title: Changes in the frequency and cost of anti-vascular endothelial therapy, and inequalities in access, in England between 2005 and 2015.

Authors: William Hollingworth[1], Tim Jones[2], Barnaby C Reeves[3], Tunde Peto[4]

1 Professor of Health Economics, School of Social and Community Medicine, University of Bristol, Bristol, BS8 2PS, United Kingdom

2 Senior Research Associate, NIHR CLAHRC West, University Hospitals Bristol NHS Foundation Trust, Bristol, BS1 2NT United Kingdom

3 Professorial Research Fellow in Health Services Research, Clinical Trials Evaluation Unit, School of Clinical Sciences, University of Bristol, Bristol, BS2 8HW, United Kingdom

4 Professor of Clinical Ophthalmology, School of Medicine, Dentistry and Biomedical Sciences, Queen's University Belfast, Belfast, BT9 7BL, United Kingdom

Corresponding author:

William Hollingworth
Professor of Health Economics
Health Economics at Bristol (HEB)
School of Social and Community Medicine
University of Bristol
Canynge Hall
39 Whatley Road
Bristol, BS8 2PS
United Kingdom

Tel: ++44 (0)117 9287355
william.hollingworth@bristol.ac.uk

Word count [3,564]

Keywords:
Vascular Endothelial Growth Factor A; Drug Costs; macular degeneration; macular edema; England; socioeconomic factors; geography

For peer review only

For peer review only - http://bmjopen.bmj.com/site/about/guidelines.xhtml

Abstract [310 words]

Objectives: High cost anti-VEGF medicines for eye disorders challenge ophthalmologists and policymakers to provide fair access for patients while minimising costs. We describe the growth in the use and costs of these medicines and measure inequalities in access.

Design: Longitudinal study using hospital episode statistics (2005/6 to 2014/15) and hospital prescribing cost reports (2008/9 to 2015/16). We used Poisson regression to estimate standardised rates and explore temporal and geographic variations.

Setting: NHS care in England.

Population: Patients receiving anti-VEGF injections for age-related macular degeneration, diabetic macular oedema and other eye disorders.

Interventions: Higher cost drugs (ranibizumab or aflibercept) recommended by NICE or lower cost drug (bevacizumab) not licensed for eye disorders.

Main outcome measures: National procedure rates and variation between and within clinical commissioning groups (CCGs). Cost of ranibizumab and aflibercept prescribing.

Results: Injection procedures increased by 215% between 2010/11 and 2014/15. In 2014/15 there were 388,031 procedures (714 per 100,000). There is no evidence that the dramatic growth in rates is slowing down. Since 2010/11 the estimated cost of ranibizumab and aflibercept increased by 247% to £447 million in 2015/16, equivalent to the entire annual budget of a CCG. There are large inequalities in access; in 2014/15 procedure rates in a ‘high use’ CCG were 9.08 times higher than in a ‘low use’ CCG. In the South West of England there was twofold variation in injections per patient per year (range 2.9 to 5.9).

Conclusions: The huge cost of anti-VEGF therapy increasingly affects the ability of the NHS to provide care for other patients. There is a lack of political and regulatory support for clinicians to use bevacizumab, which evidence suggests is more cost-effective. NICE aims to ensure that patients have equal access to cost-effective care. In this instance, it has not been able to achieve either goal.

For peer review only - http://bmjopen.bmj.com/site/about/guidelines.xhtml

Strengths and limitations

- Our study summarises new data on nationwide access to effective, but high cost ophthalmology drugs.
- Using population denominators allows us to standardise our analysis for factors, such as age and socio-economic deprivation, which will affect the need for care.
- The lack of a specific anti-VEGF drug code in HES is a limitation, however we observed similar temporal trends in two independent data sources.
- The confidential nature of drug discounts mean that our estimate of drug costs will be too high if commissioners have negotiated substantial discounts.
- Conversely, the exclusion of administration and monitoring costs make our estimates conservative.

For peer review only

For peer review only - http://bmjopen.bmj.com/site/about/guidelines.xhtml

Introduction

Clinical commissioning groups (CCGs) in England are responsible for commissioning healthcare for a geographically defined local population. On average, CCGs cover a population of 270,000 and have a budget of £300 million to commission services including elective and unplanned hospital care, community and mental health services. While CCGs have a degree of autonomy in the care they commission, they must work within national constraints. In particular, CCGs must commission new interventions which have been evaluated to be cost-effective by the National Institute for Health and Care Excellence (NICE) technology appraisal process. This presents a challenge as many of these interventions are expensive. A balance is needed in order to provide fair access for patients eligible for the new interventions without disadvantaging other patients using existing health services.

One recent challenge for CCGs is to commission anti vascular endothelial growth factor (anti-VEGF) medicines for patients with a range of eye conditions. Anti-VEGF intravitreal injections reduce new blood vessel growth and swelling [1]. Two drugs (ranibizumab and aflibercept) are licensed for use in neovascular age-related macular degeneration (nAMD), macular oedema due to diabetes (DMO) or retinal vein occlusion (RVO), and myopic choroidal neovascularisation (MCNV). Ranibizumab was first recommended by NICE as cost-effective for use in nAMD in 2008 [2]. After an initial rejection in 2011, NICE also recommended the use of ranibizumab for DMO in 2013 based on new evidence and an undisclosed discount on price offered by the manufacturer [3]. Subsequent NICE recommendations in other eye conditions and for aflibercept have followed (Table 1). Ranibizumab and aflibercept have an NHS indicative price in the region of £550 and £800 per injection, respectively [4]. The high prevalence of these conditions, the indefinite need for multiple injections per patient, and the associated procedure and monitoring costs mean that economic burden is substantial.

In reaching their recommendations, NICE did not compare ranibizumab or aflibercept with a pre-existing drug, bevacizumab. Bevacizumab also inhibits VEGF and is closely related to ranibizumab; it

For peer review only - http://bmjopen.bmj.com/site/about/guidelines.xhtml

is licensed as a treatment for certain types of cancer but not in ophthalmology. Bevacizumab can be divided into aliquots of smaller doses for ophthalmic therapy and is a much less costly alternative (approximately £50 to £100 per injection) [5]. Meta-analysis has demonstrated that bevacizumab and ranibizumab result in similar visual acuity outcomes for patients with nAMD [6]. The CATT and IVAN randomised trials of ranibizumab versus bevacizumab in nAMD both demonstrated that the drugs resulted in similar improvements in visual acuity [7, 8]. IVAN also found that ranibizumab produced little or no gain in quality-adjusted life years (QALYs) despite being substantially more expensive [9]. Individual patient data meta-analysis indicates that the risk of serious adverse events with ranibizumab and bevacizumab are similar in nAMD [10]. In DMO, the DRCR.net three-arm randomised trial found that aflibercept and ranibizumab were not cost-effective relative to bevacizumab despite evidence that aflibercept resulted in better visual outcomes at 1 and 2 years, particularly in those with worse visual acuity at baseline [11, 12]. In RVO, the SCORE2 randomised trial concluded that bevacizumab was non-inferior to aflibercept for visual acuity after 6 months of treatment [13]. In 2014, the Royal College of Ophthalmologists and others called on UK health regulators, including NICE, to urgently review the use of bevacizumab in nAMD [14]. However, in 2015 the Department of Health declined to support clinicians or commissioners using bevacizumab for nAMD, considering unlicensed use "on ground of cost alone" as potentially illegal under EU law [15], despite political support for the use of bevacizumab for ophthalmic indications in other EU countries such as Italy [16]. The remit of the forthcoming NICE nAMD clinical guidelines preclude making recommendations about bevacizumab [17].

The emergence of anti-VEGF medicines provides ophthalmologists and CCGs with a clinical and economic dilemma in both providing fair access for eligible patients to drugs recommended by NICE while minimising the opportunity costs for other members of the population. In this study, we describe the growth in the use and cost of anti-VEGF intravitreal injections in the English NHS since 2005/06. We quantify the variation in patient access to anti-VEGF therapy across England and explore the potential causes of inequality of access within the South West region of England.

For peer review only - http://bmjopen.bmj.com/site/about/guidelines.xhtml

Methods

IDENTIFYING ANTI-VEGF INJECTIONS

We used the HES admitted patient care (APC) and outpatient (OP) datasets to identify anti-VEGF injections. Anti-VEGF injections are performed in outpatient treatment rooms, but, in earlier years, were often coded in the APC dataset as day case procedures. HES is a routinely collected dataset that records all episodes of care provided to NHS funded and privately insured patients admitted (day case or inpatient) to NHS hospitals or seen at an NHS outpatient clinic [18]. HES also includes data on NHS-funded patients treated in independent sector hospitals. We extracted anonymised individual episode records on all episodes contained in the HES APC (2005/6 to 2014/15) and OP (2010/11 to 2014/15) datasets. Up to 24 clinical procedures per episode may be recorded using Office of Population, Censuses and Surveys (OPCS) (fourth revision) codes.

Anti-VEGF injection procedures are not coded consistently. The majority of healthcare providers used the code 'injection into vitreous body, not elsewhere classified [C79.4]'. In preliminary exploration of HES data we looked for negative correlations between this procedure code and other ophthalmology procedure codes to identify potential alternate procedure codes. This identified that a substantial minority used a newer code 'Injection of therapeutic substance into posterior segment of eye, not elsewhere classified [C89.3]'. HES does not record the specific drug injected into the eye; however, since 2009 an OPCS code for high cost subfoveal choroidal neovascularisation drugs Band 1 [X93.1] has been in use. This code may be used for aflibercept, bevacizumab, ranibizumab, and two other drugs rarely used in intravitreal injections, pegaptanib and verteporfin. We excluded any procedures using non anti-VEGF drugs (X93.2, X93.3).

In 2014/15, 73.3% (284,490/388,031) of episodes including an eye injection procedure also coded a high cost subfoveal choroidal neovascularisation drug. However, some hospitals performing hundreds of intravitreal injections each year never use this high cost drug code. Conversely, 96%

For peer review only - http://bmjopen.bmj.com/site/about/guidelines.xhtml

(284,490/296,129) of episodes coding a high cost subfoveal choroidal neovascularisation drug also included an eye injection procedure (Figure 1). In our analysis, we assumed that procedures coded as C79.4 or C89.3 were anti-VEGF injections. We explore the importance of this assumption in our discussion.

A minority of patients receive bilateral injections on the same day, which may be coded using the supplemental 'bilateral operation' [Z94.1] OPCS code. When a patient had a bilateral procedure coded, we counted this as two injection procedures. When a patient had two injection procedures coded on the same day without any supplemental bilateral code, we assumed that this also represented bilateral procedures. If patients had more than two injection procedures coded on the same day, we excluded the 'excess' procedures as probable coding error duplicates (<0.03% of all procedures).

We used ICD-10 diagnosis codes to identify patients with diabetes with ophthalmic complications (E10.3, E11.3, E12.3, E13.3, or E14.3) or diabetic retinopathy (H36.0) or with a primary code indicating “other specified retinal disorders” (H35.8) combined with secondary codes indicating diabetes with ophthalmic complications as above; nAMD (H35.3); RVO (H34.8); and MCNV (H44.2) respectively. Diagnosis codes are well recorded for procedures in the HES APC dataset, however in the HES OP dataset diagnosis codes are often missing. Therefore, we limited our analysis of diagnostic indications to procedures recorded in the HES APC dataset.

ESTIMATING PROCEDURE RATES

National trends over time were estimated using directly standardised procedure rates [19] (per 100,000 population), with the population of England in 2014 as our standard population. We first summed the number of anti-VEGF injections, grouped by sex, quintiles of age, and financial year. These procedure counts were used to calculate annual age-sex-specific rates, by dividing by the appropriate age-sex-specific mid-year populations of England [20] (e.g. for the 2012/13 financial

For peer review only - http://bmjopen.bmj.com/site/about/guidelines.xhtml

year, the mid-2012 populations were used). We weighted the annual age-sex-specific rates according to the population distribution of England in 2014, to produce directly standardised rates for each year.

Patients typically have more than one injection procedure in a year. Variation in patient access to anti-VEGF injections within England might be evident in both the number of patients treated and the number of procedures performed. Therefore, we estimated indirectly standardised rates [19] per 100,000 population for both patients and procedures. We first calculated age-sex-specific rates for England in 2014/15, then multiplied these rates by the age-sex-specific population for the area of interest [20-22] (e.g. CCG) and summed the results. This produced the expected number of patients and procedures for that area, if it were to have the same age-sex-specific rates as England. The expected number was then compared to the observed number of patients and procedures for that area. A Poisson regression model was fitted to the observed counts, with the expected counts as an offset and socio-economic deprivation (using the overall score from the English Indices of Multiple Deprivation[23]) and ethnicity (% white British[24]) as predictive factors. The model was then used to predict new expected counts for each area based on deprivation and ethnicity, and form indirectly standardised patient and procedure ratios (observed / expected). Statistical analyses were conducted using Stata/MP 14.2 for Windows and we mapped variation in patient and procedure rates across England in 2014/15 using ArcGIS ArcMap 10.3.1 for Desktop.

EXPLORING VARIATION IN ACCESS IN THE SOUTH WEST REGION OF ENGLAND

For each CCG in the South West region of England we estimated the median age and gender proportion in order to explore any differences in the demographic profile of patients receiving anti-VEGF injections. We estimated the annual number of procedures per patient in 2014/15 to determine whether CCGs with high patient rates also had a higher number of procedures per patient. We also calculated and mapped indirectly standardised procedure ratios between Middle

For peer review only - http://bmjopen.bmj.com/site/about/guidelines.xhtml

Layer Super Output Areas (MSOA) to explore whether CCG boundaries or hospital catchment areas were more strongly associated with access to anti-VEGF injections.

ESTIMATING THE COST OF ANTI-VEGF INJECTIONS

We extracted data from NHS Digital Hospital prescribing costs reports [25] in order to estimate the costs of ranibizumab and aflibercept since 2008/9 when ranibizumab was first approved by NICE. The NHS Digital report summarises data collected from English NHS hospital pharmacies by the IMS Health Hospital Pharmacy Audit Index (HPAI). This includes high cost medicines supplied to wards, outpatient clinics and satellite sites. Costs are reported in the HPAI and are calculated by IMS Health using standard price lists and therefore do not necessarily reflect any discounted prices paid by hospitals. Anti-VEGF therapy also incurs administration and monitoring costs; however, the IVAN trial indicated that, in the ranibizumab group, drug costs accounted for 80-88% of total costs [9]. Injection frequency and monitoring visits may be lower for aflibercept where fixed 2-monthly interval dosing is recommended after an initial period of monthly loading doses.

Bevacizumab is not included in the HPAI and due to the lack of drug coding in HES data it is impossible to accurately estimate the current use of bevacizumab for eye conditions. A study in 2015 based on 189 Freedom of Information requests across the UK estimated the relative prevalence of injections of ranibizumab, aflibercept and bevacizumab to be 61.1%, 36.0% and 2.8% respectively [26].

For peer review only - http://bmjopen.bmj.com/site/about/guidelines.xhtml

Results

THE USE AND COST OF ANTI-VEGF INTRAVITREAL INJECTIONS

In 2014/15 there were 388,031 intra-vitreal injection procedures, equivalent to 714 procedures per 100,000 population (Figure 2). This compares to 123,006 procedures in 2010/11 and represents a 215% increase in the number of procedures over the five-year period. Before 2007, intra-vitreal injections were rarely used. The rise in procedure rates since then is dramatic and there are no signs that it is levelling off.

Amongst episodes recorded in the HES APC dataset, the majority (62% in 2014/15) had diagnosis codes indicating nAMD (Figure 3). The relative use of diagnosis codes indicating DMO or RVO show small recent increases associated with NICE approvals for these indications in 2013. The estimated total cost of hospital prescribing of ranibizumab and aflibercept increased by 197% from £129 million to £383 million in the five years between 2010/11 and 2014/15 (Figure 4). The estimated total cost of ranibizumab and aflibercept had risen to £447 million in 2015/16, predominantly due to the increasing use of aflibercept.

VARIATION IN PATIENT ACCESS TO ANTI-VEGF THERAPY

In 2014/15 there was substantial variation in procedure rates between CCGs, even after adjusting for age, sex, deprivation, and ethnicity profiles (Figure 5a). The map demonstrates pockets of very high use (>150% times the expected rate), for example in the Bristol area and in parts of North London and Norfolk. There are also areas where procedure rates are less than 50% of the expected rate, for example in parts of Essex. There was less pronounced, but still substantial variation in patient rates between CCGs, after adjusting for age, sex, deprivation and ethnicity profiles (Figure 5b). Areas of the country with high procedure rates also tended to have higher proportions of their populations having treatment. In 2014/15 the ratio in patient rates between a 'high use' CCG at the 90th percentile and a 'low use' CCG at the 10th percentile was 4.98. This ratio has only slightly decreased

For peer review only - http://bmjopen.bmj.com/site/about/guidelines.xhtml

since 2010/11, when the ratio was 6.07, indicating that equality of access to care is only slowly improving over time.

Examining the South West region of England in more detail (Figures 6a and 6b), it is evident that there is substantial variation in access to anti-VEGF injections within CCGs. For example, in both Somerset and Wiltshire there are areas of relatively high procedure rates closer to the major conurbations of Bath, Bristol and Swindon. Procedure rates are much lower than expected in other areas of these CCGs, even though these other areas are served by local hospitals. The age and gender profile of patients receiving anti-VEGF therapy was similar between CCGs (Table 2). Median age ranged from 77 to 82 years and between 39% and 49% of patients were male. There was twofold variation in the number of procedures per patient per year ranging from 2.9 in Cornwall to 5.9 in South Gloucestershire. The standardised CCG patient rate was strongly correlated with the number of procedures per patient (Spearman's Rho = 0.71, $p < .001$).

For peer review only

For peer review only - http://bmjopen.bmj.com/site/about/guidelines.xhtml

Discussion

STATEMENT OF PRINCIPAL FINDINGS

There is no evidence that the dramatic growth in the use of high cost anti-VEGF injections in ophthalmology is slowing down. Injection procedures increased by 215% between 2010/11 and 2014/15. There are large inequalities in access to anti-VEGF therapy. This inequality is not explained by differences in demographics and occurs both within and between CCGs. Procurement of high cost anti-VEGF drugs has placed a large and increasing strain on the NHS. In 2015/16, the NHS spent an estimated £447 million on drug costs alone, equivalent to the entire annual budget of a large CCG.

STRENGTHS AND WEAKNESSES OF THE STUDY

Our study summarises new data on nationwide access to effective, but high cost ophthalmology drugs. Using population denominators allows us to standardise our analysis for factors, such as age and socio-economic deprivation, which will affect the need for care. HES data records patients' area of residence, so we compare access based on place of residence rather than place of treatment. The combination of HES APC and OP datasets is essential in order to accurately monitor intravitreal injections over time. As reimbursement payments depend on procedure coding, there is an incentive for providers to fully code procedures. However, the lack of a specific anti-VEGF drug code in HES is a limitation. Other similar procedures such as the intravitreal injection of dexamethasone implants have distinct OPCS procedure codes, although we cannot exclude the possibility of miscoding. We observed similar temporal trends in two independent data sources measuring procedures (i.e. HES) and drug costs (i.e. HPAI) which provides reassurance about the validity of our time-trend analyses. Previous work has demonstrated that HES generally has good sensitivity in identifying new procedures, but may lack specificity particularly in the absence of a single specific procedure code [27]. It is plausible that discounts account for some of the observed variations, if

For peer review only - http://bmjopen.bmj.com/site/about/guidelines.xhtml

CCGs who have larger discounts offer more therapy. The confidential nature of drug discounts mean that our estimate of drug costs will be too high if CCGs have negotiated substantial discounts. Conversely, the exclusion of administration and monitoring costs make our estimates conservative and there is a substantial economic burden for patients. Travel and post-appointment recovery time have a large impact on patients and carers [28]. The costs of all three anti-VEGF drugs will be offset, to a greater or lesser extent, in the longer term by reduced need for the NHS and social services related to vision loss.

COMPARISON WITH OTHER STUDIES

Between 1989/90 and 2008/9, Keenan et al observed an increase in intravitreal injection rates from 0.4 per 100,000 to 59.5 per 100.000 with wide variations in access between different local authorities in England [29]. Our data demonstrate that this rapid growth has continued and even accelerated in the following six years. We were also able to explore variation in access to care at a more granular level and demonstrate that access varies markedly both within and between CCGs, suggesting that variations are likely to be due, at least in part, to the availability of trained staff (e.g. ophthalmologists and nurse practitioners) and facilities in local hospitals rather than CCG-wide policies. Using insurance data, Parikh et al observed a continually increasing trend in anti-VEGF use in the US between 2006 and 2015 [30]. In line with our study, Parikh et al also found that anti-VEGF drug use was predominantly for nAMD. It is impossible to directly compare England and US rates as the denominator used in the Parikh et al study excludes patients without commercial or Medicare Advantage insurance. However, anti-VEGF therapy rates in the US and UK appear to be of the same order of magnitude. Specific coding of anti-VEGF drugs allowed Parikh et al to document the rise in the use of aflibercept (28% of all anti-VEGF injections in 2015) and the concurrent levelling off in the use of ranibizumab (17%) and bevacizumab (55%). This is in stark contrast to England where ranibizumab and aflibercept predominate largely as a result of legal challenges to the use of bevacizumab and the lack of Department of Health support for commissioners [31].

For peer review only - http://bmjopen.bmj.com/site/about/guidelines.xhtml

IMPLICATIONS FOR POLICYMAKERS AND CLINICIANS

Based on the IVAN trial, Dakin et al estimated that the NHS could save £102 million per year by switching from ranibizumab to bevacizumab [9]. In the US, Hutton et al estimated that a similar switch would save Medicare $18 billion over a 10-year period [32]. Given the lack of political and regulatory support for clinicians to use bevacizumab, it is unsurprising that most in England do not use it. This has led to the very unusual situation whereby the NHS is paying for more expensive therapy than healthcare insurers in the US. The patent on ranibizumab is due to expire in Europe in 2022 [33]; in the meantime, based on current trends, the NHS may spend billions on anti-VEGF injections, before biosimilar drugs become available. Our data, demonstrating large variations between CCGs in the numbers of patients accessing anti-VEGF services also suggests that there is considerable potential unmet need in some areas of the country. Given that many CCGs are already in financial deficit, most will struggle to afford to treat more patients unless they are able to switch to bevacizumab. The political decision not to support NHS use of bevacizumab in eye conditions is in stark contrast to decisions taken in other EU countries [16] and has very large negative consequences for NHS patients.

In April 2017, NICE introduced a budget impact test for new technologies under appraisal that are expected to cost more than £20 million per annum. This allows NHS England up to three years to conduct commercial negotiations on price rather than the current requirement to fund within 90 days [34]. This policy should strengthen the future negotiating power of the NHS for expensive drugs. However, it does not address the fundamental problem that NICE has not included cost-effective but unlicensed drugs in technology appraisals or clinical guideline recommendations.

The widespread variation in procedure rates that we observed might be due to travel distance affecting patient access to diagnostic and therapeutic services, particularly in more remote areas. Lengthy travel distance is associated with treatment discontinuance [35]. However, many of the low injection rate areas in the South West of England are close to hospitals. Other unmeasured factors,

For peer review only - http://bmjopen.bmj.com/site/about/guidelines.xhtml

such as the importance of vision for work or usual activities, may also be associated with patient uptake and compliance with anti VEGF therapy. The frequency of injections per patient per year was lower than reported in trials of therapy initiation. The averages in our study will be drawn downwards due to patients starting and discontinuing therapy partway through the year and by patients who have been on anti-VEGF therapy for many years and need fewer injections.

UNANSWERED QUESTIONS AND FUTURE RESEARCH

Given the high cost of the drugs, it is imperative that clinical services are organised to deliver care efficiently. For example, nurse-administered injections [36], treat and extend protocols [37], optometrist-led monitoring [38] and stereotactic radiotherapy [39] all offer the potential to reduce costs and require stronger evidence. The wide variation between CCGs in the number of injections per patient represents a natural experiment. Observational studies could compare costs and outcomes between areas of the country with high and low injection frequency. However, the lack of a nationwide register of patients receiving anti-VEGF injections and the lack of drug coding in hospital episode statistics severely limit the potential to conduct this research.

CONCLUSIONS

Anti-VEGF therapy is effective in most cases and has improved the quality of life of many thousands of patients in England. However, the lack of Government and regulatory support for the use of bevacizumab, a cheaper medication with proven efficacy, limits the ability of the NHS to pay for care for other patients. NICE was established in 1999 to ensure that patients in England have equal access to cost-effective care. In this instance, it has not been able to achieve either goal.

For peer review only - http://bmjopen.bmj.com/site/about/guidelines.xhtml

Acknowledgements:
Hospital Episode Statistics were provided by NHS Digital under data sharing agreement (NIC-17875-X7K1V) with the University of Bristol. Copyright © 2016, re-used with the permission of The Health & Social Care Information Centre. All rights reserved.

Funding:
William Hollingworth and Tim Jones receive funding from the National Institute for Health Research Collaboration for Leadership in Applied Health Research and Care (NIHR CLAHRC) West. The views expressed are those of the authors and not necessarily those of the NHS, the NIHR or the Department of Health. The funder had no role in the study design, collection, analysis and interpretation of data or in the writing of the report and the decision to submit the article for publication.

For peer review only

Competing Interests:
All authors have completed the Unified Competing Interest form (available on request from the corresponding author) and declare: no support from any organisation for the submitted work; no financial relationships with any organisations that might have an interest in the submitted work in the previous three years, no other relationships or activities that could appear to have influenced the submitted work.

Copyright statement:
The Corresponding Author has the right to grant on behalf of all authors and does grant on behalf of all authors, a worldwide licence to the Publishers and its licensees in perpetuity, in all forms, formats and media (whether known now or created in the future), to i) publish, reproduce, distribute, display and store the Contribution, ii) translate the Contribution into other languages, create adaptations, reprints, include within collections and create summaries, extracts and/or, abstracts of the Contribution, iii) create any other derivative work(s) based on the Contribution, iv) to exploit all subsidiary rights in the Contribution, v) the inclusion of electronic links from the Contribution to third party material where-ever it may be located; and, vi) licence any third party to do any or all of the above.

We attest that we have obtained appropriate permissions and paid any required fees for use of copyright protected materials.

Transparency declaration:
The lead author affirms that the manuscript is an honest, accurate, and transparent account of the study being reported; that no important aspects of the study have been omitted; and that any discrepancies from the study as planned (and, if relevant, registered) have been explained.

Details of contributors:
WH initiated and designed the study, contributed to the data analysis and interpretation of data and drafted and revised the paper. He is guarantor. TJ contributed to the study design, cleaned and analysed the data, contributed to the interpretation of data and revised the draft paper. BR and TP contributed to the interpretation of data and revised the draft paper. All authors approved the final version of the paper. All authors had full access to all of the data in the study and can take responsibility for the integrity of the data and the accuracy of the data analysis.

Ethical approval:

The paper describes work based on anonymised individual episode records provided under a data sharing agreement between NHS Digital and the University of Bristol. The data were not linked to other datasets or re-identified. Ethical approval was not required.

Data sharing:
No additional data available.

For peer review only

For peer review only - http://bmjopen.bmj.com/site/about/guidelines.xhtml

Figures

Figure 1 – OPCS codes used to identify anti-VEGF injections: 2014/15

Note: 2,479 eye injection procedures were excluded due to combination with X93.2 or X93.3 high-cost drug codes.

Figure 2. Directly-standardised intravitreal injection procedure rates, England, 2005/06-2014/15

Note: OP = Outpatient; APC = Admitted Patient Care

Figure 3. Percentage of total eye injection procedures in HES APC for different primary diagnoses, England, 2008/09-2014/15.

Note: nAMD = Neovascular Age-Related Macular Degeneration; DMO = Diabetic Macular Oedema; RVO = Retinal Vein Occlusion; Due to very small numbers of procedures for Myopic Choroidal Neo-Vascularisation (<=10 per year), this was included in 'Other'.

Figure 4. Hospital prescribing costs for Ranibizumab and Aflibercept, 2008/09 to 2015/16

Figure 5a. Indirectly age-sex-deprivation-ethnicity standardised procedure ratios for combined eye injection procedures (OPCS-4: C794 and C893) by CCG in England, 2014/15.

Figure 5b. Indirectly age-sex-deprivation-ethnicity standardised patient ratios for combined eye injection procedures (OPCS-4: C794 and C893) by CCG in England, 2014/15.

Figure 6a. Indirectly standardised procedure ratios for combined eye injection procedures by MSOA in SW England, 2014/15

Note: Major hospitals marked including those in NHS Trusts providing 100+ procedures.

Figure 6b. Indirectly standardised patient ratios for combined eye injection procedures by MSOA in SW England, 2014/15

Note: Major hospitals marked including those in NHS Trusts providing 100+ procedures.

For peer review only - http://bmjopen.bmj.com/site/about/guidelines.xhtml

Table 1 – NICE appraisals for anti-VEGF therapies

Condition	Ranibizumab	Aflibercept
nAMD	Recommend 8/2008 [TA 155]	Recommend 7/2013 [TA 294]
DMO	Reject 11/2011 [TA 237]<br>Recommend 2/2013 [TA 274]	Recommend 7/2015 [TA 346]
RVO	Recommend 5/2013 [TA 294]	Recommend 2/2014 [TA 305]
MCNV	Recommend 11/2013 [TA 298]	Recommend 9/2016 [TA 409]

For peer review only

For peer review only - http://bmjopen.bmj.com/site/about/guidelines.xhtml

Table 2. Demographics of anti-VEGF procedures in South West CCGs, 2014/15; ordered by procedures per patient.

CCG Name	% Male	Median Age	Procedures per Patient (95% CI)	Procedure Rate* (95% CI**)	Patient Rate* (95% CI)
NHS Kernow CCG	43	79	2.86 (2.75-2.97)	522 (490-556)	172 (191-104)
NHS Dorset CCG	43	82	3.32 (3.21-3.43)	419 (394-445)	122 (136-74)
NHS Herefordshire CCG	38	80	3.34 (3.14-3.53)	631 (569-700)	175 (210-110)
NHS Somerset CCG	45	78	3.48 (3.27-3.68)	304 (276-334)	92 (106-57)
NHS South Warwickshire CCG	44	79	3.63 (3.46-3.8)	773 (708-844)	206 (240-128)
NHS Oxfordshire CCG	40	81	3.63 (3.5-3.76)	723 (679-771)	197 (220-120)
NHS Northern Eastern and Western Devon CCG	41	81	4.05 (3.95-4.16)	830 (792-869)	201 (218-121)
NHS Newbury and District CCG	41	79	4.14 (3.72-4.55)	637 (530-764)	136 (186-92)
NHS Wiltshire CCG	46	79	4.17 (3.95-4.39)	459 (419-502)	103 (121-64)
NHS South Devon and Torbay CCG	40	81	4.2 (4.05-4.35)	923 (860-991)	206 (235-127)
NHS South Worcestershire CCG	42	79	4.36 (4.16-4.56)	875 (806-950)	188 (217-116)
NHS Bath and North East Somerset CCG	49	81	4.44 (4.14-4.73)	735 (650-831)	152 (189-97)
NHS Swindon CCG	44	77	4.54 (4.26-4.82)	1034 (928-1153)	216 (260-137)
NHS West Hampshire CCG	42	80	5 (4.85-5.15)	1125 (1063-1190)	221 (244-133)
NHS North Somerset CCG	43	80	5.43 (5.15-5.71)	1236 (1130-1352)	217 (253-135)
NHS Bristol CCG	43	80	5.49 (5.26-5.72)	1346 (1248-1451)	237 (270-146)
NHS Gloucestershire CCG	42	81	5.51 (5.33-5.68)	1263 (1194-1336)	226 (249-137)
NHS South Gloucestershire CCG	46	79	5.93 (5.65-6.22)	1379 (1264-1505)	222 (258-138)

** Procedure rate and patient rate are the indirectly standardised rates per 100,000 population*

*** 95% CIs on the procedure rate are calculated using Stukel et al.'s method for recurrent outcomes[40]*

For peer review only

For peer review only - http://bmjopen.bmj.com/site/about/guidelines.xhtml

References

1. Steinbrook, R., *The price of sight--ranibizumab, bevacizumab, and the treatment of macular degeneration.* N Engl J Med, 2006. **355**(14): p. 1409-12.
2. National Institute for Health and Care Excellence, *Ranibizumab and pegaptanib for the treatment of age-related macular degeneration*. 2008.
3. National Institute for Health and Care Excellence, *Ranibizumab for treating diabetic macular oedema*. 2013.
4. BMJ Group and Pharmaceutical Press, *British National Formulary*. 2017: London.
5. Poku, E., et al., *Bevacizumab in eye conditions: Issues related to quality, use, efficiency and safety*. 2012, NICE Decision Support Unit: Sheffield.
6. Solomon, S.D., et al., *Intravitreal Bevacizumab Versus Ranibizumab for Treatment of Neovascular Age-Related Macular Degeneration: Findings from a Cochrane Systematic Review.* Ophthalmology, 2016. **123**(1): p. 70-77 e1.
7. Chakravarthy, U., et al., *Alternative treatments to inhibit VEGF in age-related choroidal neovascularisation: 2-year findings of the IVAN randomised controlled trial.* Lancet, 2013. **382**(9900): p. 1258-67.
8. Comparison of Age-related Macular Degeneration Treatments Trials Research, G., et al., *Ranibizumab and bevacizumab for treatment of neovascular age-related macular degeneration: two-year results.* Ophthalmology, 2012. **119**(7): p. 1388-98.
9. Dakin, H.A., et al., *Cost-effectiveness of ranibizumab and bevacizumab for age-related macular degeneration: 2-year findings from the IVAN randomised trial.* BMJ Open, 2014. **4**(7): p. e005094.
10. Maguire, M., et al., *Serious Adverse Events with Bevacizumab or Ranibizumab for Age-Related Macular Degeneration: Meta-analysis of Individual Patient Data.* Ophthalmology Retina, In Press.
11. Ross, E.L., et al., *Cost-effectiveness of Aflibercept, Bevacizumab, and Ranibizumab for Diabetic Macular Edema Treatment: Analysis From the Diabetic Retinopathy Clinical Research Network Comparative Effectiveness Trial.* JAMA Ophthalmol, 2016. **134**(8): p. 888-96.
12. Wells, J.A., et al., *Aflibercept, Bevacizumab, or Ranibizumab for Diabetic Macular Edema: Two-Year Results from a Comparative Effectiveness Randomized Clinical Trial.* Ophthalmology, 2016. **123**(6): p. 1351-9.
13. Scott, I.U., et al., *Effect of Bevacizumab vs Aflibercept on Visual Acuity Among Patients With Macular Edema Due to Central Retinal Vein Occlusion: The SCORE2 Randomized Clinical Trial.* JAMA, 2017. **317**(20): p. 2072-2087.
14. Lotery, A. and C. MacEwen, *What is stopping the NHS from using bevacizumab for macular degeneration and other retinal disorders?* BMJ, 2014. **349**: p. g6887.
15. Freeman, G., *Letter to NHS Southern Derbyshire CCG*, Department of Health, Editor. 2015.
16. Formoso, G., et al., *A clearer view of evidence in treating macular degeneration: off-label policies and independent research.* Cochrane Database Syst Rev, 2014(9): p. ED000090.
17. National Institute for Health and Care Excellence, *Guideline scope age-related macular degeneration: diagnosis and management* 2015, NICE.
18. Health and Social Care Information Centre, *Hospital Episode Statistics (HES) Analysis Guide*. 2015.
19. Anon, *Standardization: a classic epidemiological method for the comparison of rates.* Epidemiological Bulletin, 2002. **23**(3): p. 9-12.
20. Office for National Statistics. *Lower Super Output Area Mid-Year Population Estimates* [cited 2016; Available from: https://www.ons.gov.uk/peoplepopulationandcommunity/populationandmigration/populationestimates/datasets/lowersuperoutputareamidyearpopulationestimates.

For peer review only - http://bmjopen.bmj.com/site/about/guidelines.xhtml

21. Office for National Statistics. *Lower Layer Super Output Area (2011) to Clinical Commissioning Group to Local Authority District (July 2015) Lookup in England*. [cited 2016; Available from: http://ons.maps.arcgis.com/home/item.html?id=7de88cd2369d4006925de49e8f3f2e27.
22. Office for National Statistics. *Output Area to Lower Layer Super Output Area to Middle Layer Super Output Area to Local Authority District (December 2011) Lookup in England and Wales*. [cited 2016; Available from: http://ons.maps.arcgis.com/home/item.html?id=baf6566ca08949c6bbd61ff81d9526da.
23. Government, D.o.C.a.L. *English Indices of Deprivation 2015*. [cited 2016; Available from: https://www.gov.uk/government/statistics/english-indices-of-deprivation-2015.
24. Nomis; Office for National Statistics. *Ethnic group of the usual resident population of England and Wales as at census day, 27 March 2011*. [cited 2016; Available from: https://www.nomisweb.co.uk/census/2011/ks201ew.
25. NHS Digital, *Prescribing Costs in Hospitals and the Community*. 2016.
26. Di Simplicio, S., et al. *A UK survey of anti-VEGF use for the eye in January 2015*. in *The Association for Research in Vision and Ophthalmology Annual Meeting*. 2017. Baltimore, MD.
27. Patrick, H., et al., *Monitoring the use and outcomes of new devices and procedures: how does coding affect what Hospital Episode Statistics contribute? Lessons from 12 emerging procedures 2006-10.* J Public Health (Oxf), 2013. **35**(1): p. 132-8.
28. Prenner, J.L., et al., *Disease Burden in the Treatment of Age-Related Macular Degeneration: Findings From a Time-and-Motion Study.* Am J Ophthalmol, 2015. **160**(4): p. 725-31 e1.
29. Keenan, T.D., C.J. Wotton, and M.J. Goldacre, *Trends over time and geographical variation in rates of intravitreal injections in England.* Br J Ophthalmol, 2012. **96**(3): p. 413-8.
30. Parikh, R., et al., *Trends of Anti-Vascular Endothelial Growth Factor Use in Ophthalmology Among Privately Insured and Medicare Advantage Patients.* Ophthalmology, 2016.
31. Cohen, D., *Why have UK doctors been deterred from prescribing Avastin?* BMJ, 2015. **350**: p. h1654.
32. Hutton, D., et al., *Switching to less expensive blindness drug could save medicare part B $18 billion over a ten-year period.* Health Aff (Millwood), 2014. **33**(6): p. 931-9.
33. Generics and biosimilars initiative. *Biosimilars of ranibizumab*. 2015 18 May 2017]; Available from: http://gabionline.net/Biosimilars/General/Biosimilars-of-ranibizumab.
34. Charlton, V., et al., *Cost effective but unaffordable: an emerging challenge for health systems.* BMJ, 2017. **356**: p. j1402.
35. Boulanger-Scemama, E., et al., *Ranibizumab for exudative age-related macular degeneration: A five year study of adherence to follow-up in a real-life setting.* J Fr Ophtalmol, 2015. **38**(7): p. 620-7.
36. Austeng, D., et al., *Nurse-administered intravitreal injections of anti-VEGF: study protocol for noninferiority randomized controlled trial of safety, cost and patient satisfaction.* BMC Ophthalmol, 2016. **16**(1): p. 169.
37. Chin-Yee, D., et al., *A systematic review of as needed versus treat and extend ranibizumab or bevacizumab treatment regimens for neovascular age-related macular degeneration.* Br J Ophthalmol, 2016. **100**(7): p. 914-917.
38. Violato, M., et al., *Cost-effectiveness of community versus hospital eye service follow-up for patients with quiescent treated age-related macular degeneration alongside the ECHoES randomised trial.* BMJ Open, 2016. **6**(10): p. e011121.
39. Neffendorf, J.E., et al., *StereoTactic radiotherapy for wet Age-Related macular degeneration (STAR): study protocol for a randomised controlled clinical trial.* Trials, 2016. **17**(1): p. 560.
40. Stukel, T.A., et al., *Standardized Rates of Recurrent Outcomes.* Statistics in Medicine, 1994. **13**(17): p. 1781-1791.

For peer review only - http://bmjopen.bmj.com/site/about/guidelines.xhtml

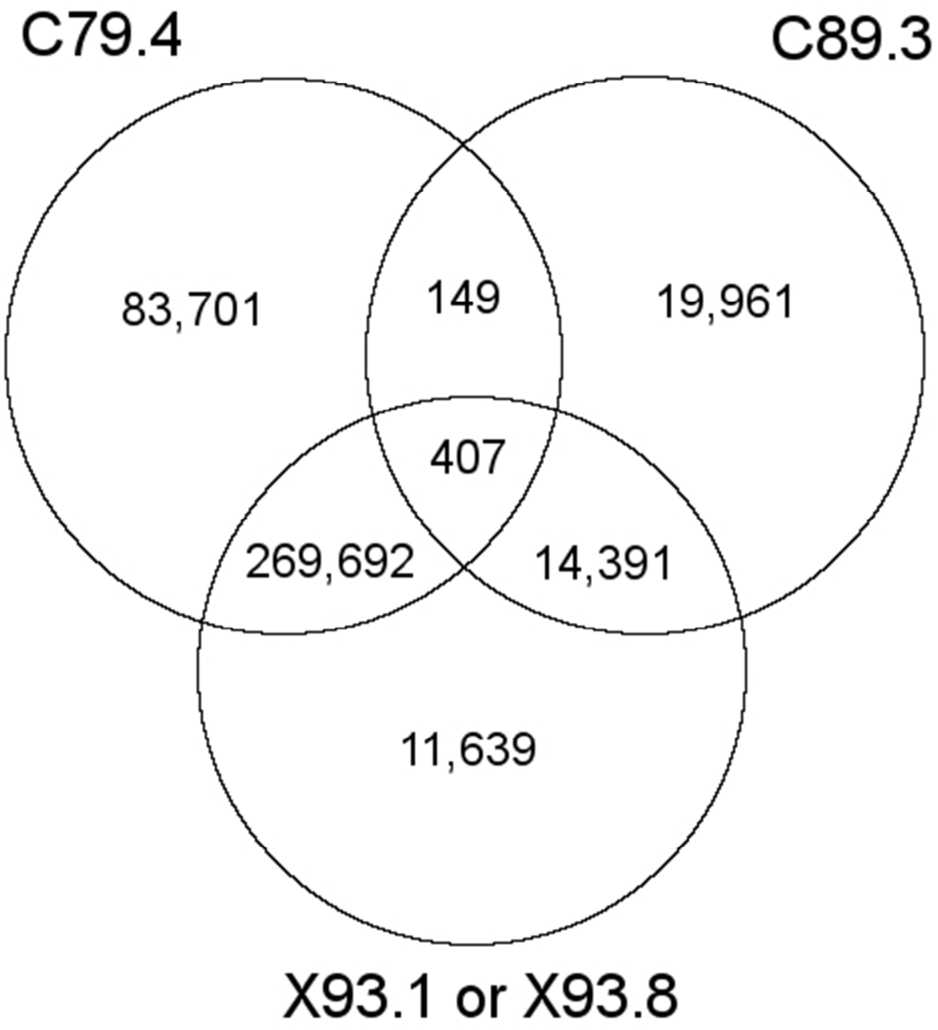

Figure 1 – OPCS codes used to identify anti-VEGF injections: 2014/15
Note: 2,479 eye injection procedures were excluded due to combination with X93.2 or X93.3 high-cost drug codes.

40x46mm (300 x 300 DPI)

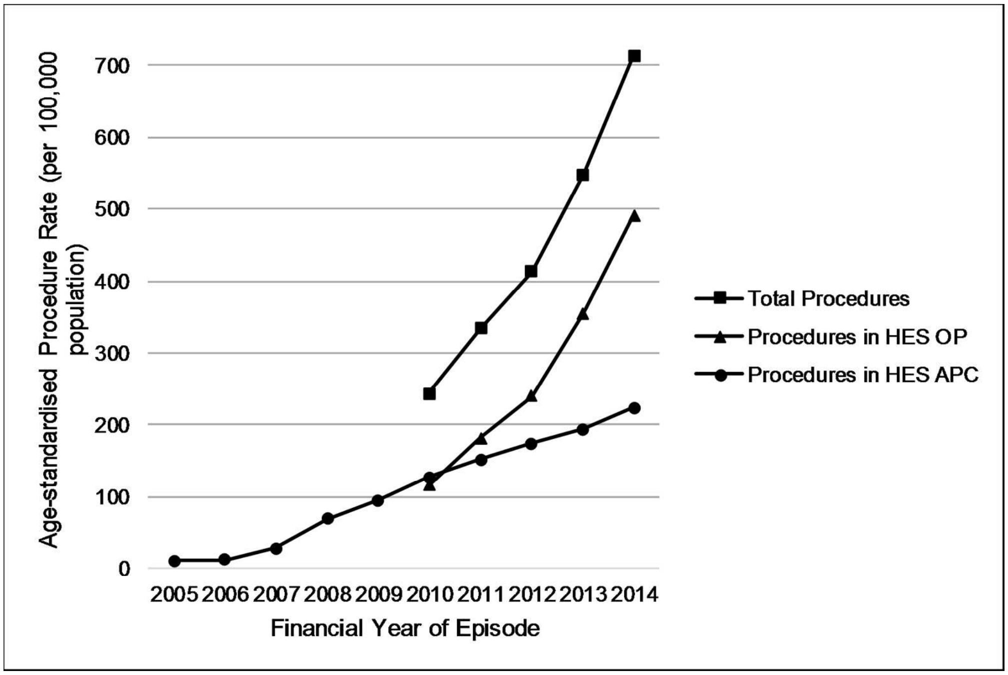

Figure 2. Directly-standardised intravitreal injection procedure rates, England, 2005/06-2014/15
Note: OP = Outpatient; APC = Admitted Patient Care

148x98mm (300 x 300 DPI)

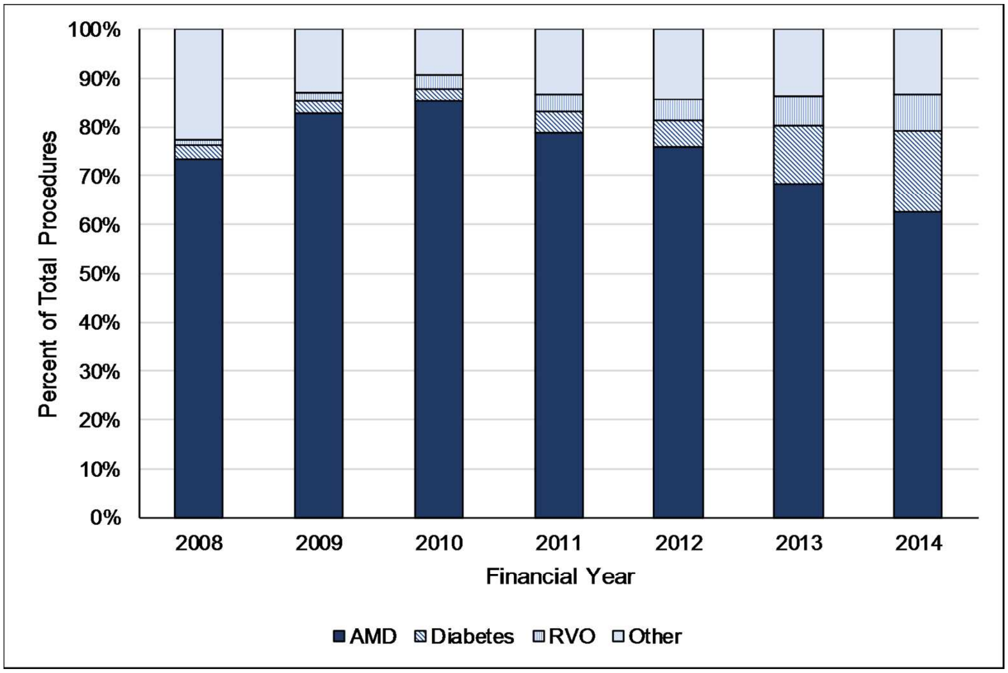

Figure 3. Percentage of total eye injection procedures in HES APC for different primary diagnoses, England, 2008/09-2014/15.
Note: nAMD = Neovascular Age-Related Macular Degeneration; DMO = Diabetic Macular Oedema; RVO = Retinal Vein Occlusion; Due to very small numbers of procedures for Myopic Choroidal Neo-Vascularisation (<=10 per year), this was included in ‘Other’.

150x99mm (300 x 300 DPI)

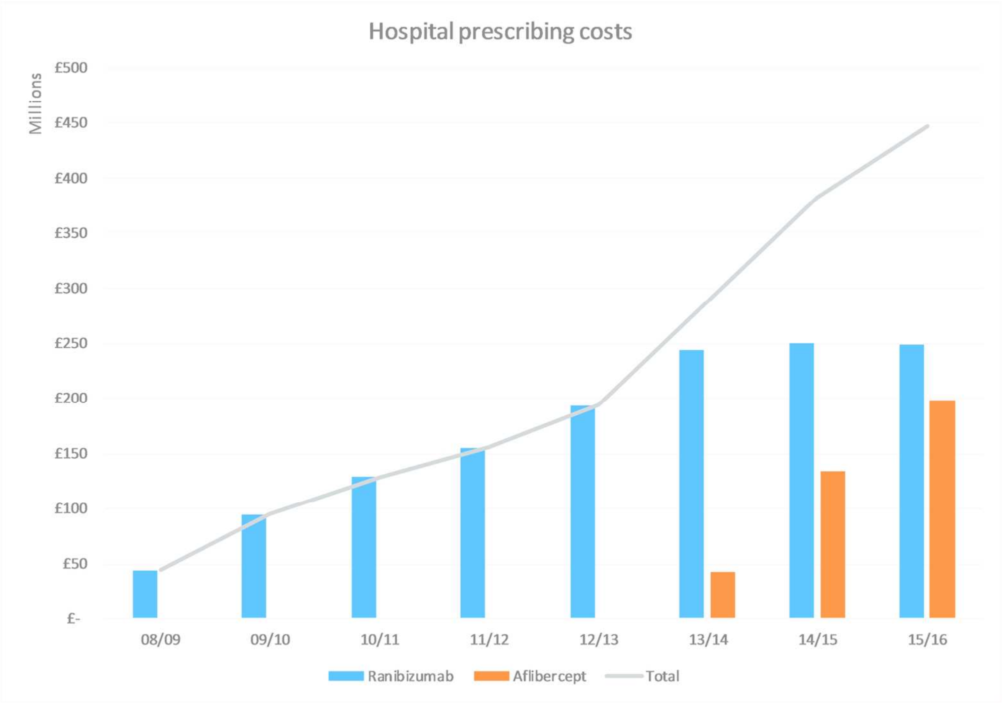

Figure 4. Hospital prescribing costs for Ranibizumab and Aflibercept, 2008/09 to 2015/16

136x95mm (300 x 300 DPI)

For peer review only

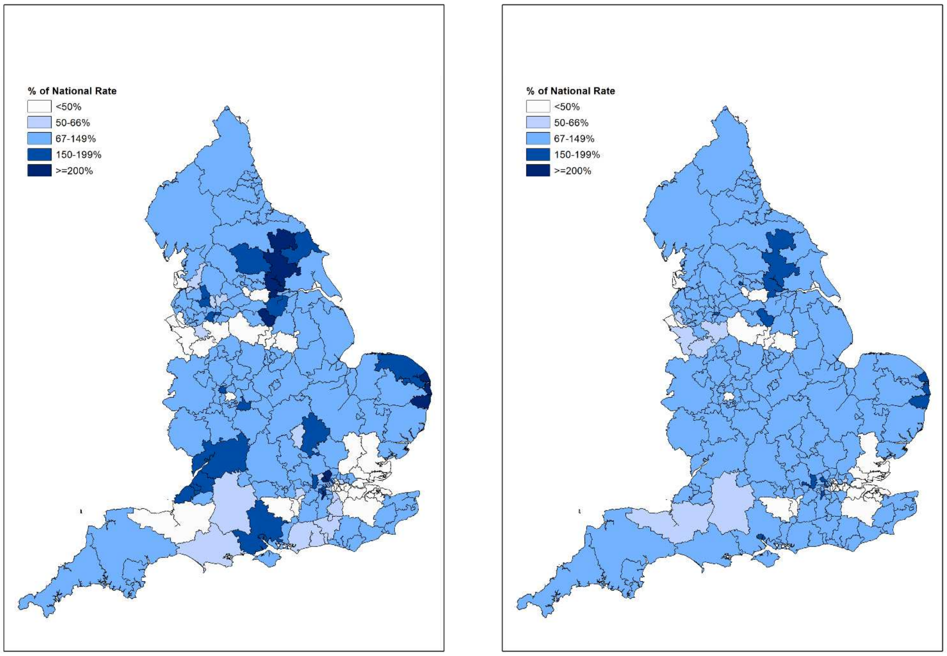

Figure 5a. Indirectly age-sex-deprivation-ethnicity standardised procedure ratios for combined eye injection procedures (OPCS-4: C794 and C893) by CCG in England, 2014/15.
Figure 5b. Indirectly age-sex-deprivation-ethnicity standardised patient ratios for combined eye injection procedures (OPCS-4: C794 and C893) by CCG in England, 2014/15.

420x296mm (300 x 300 DPI)

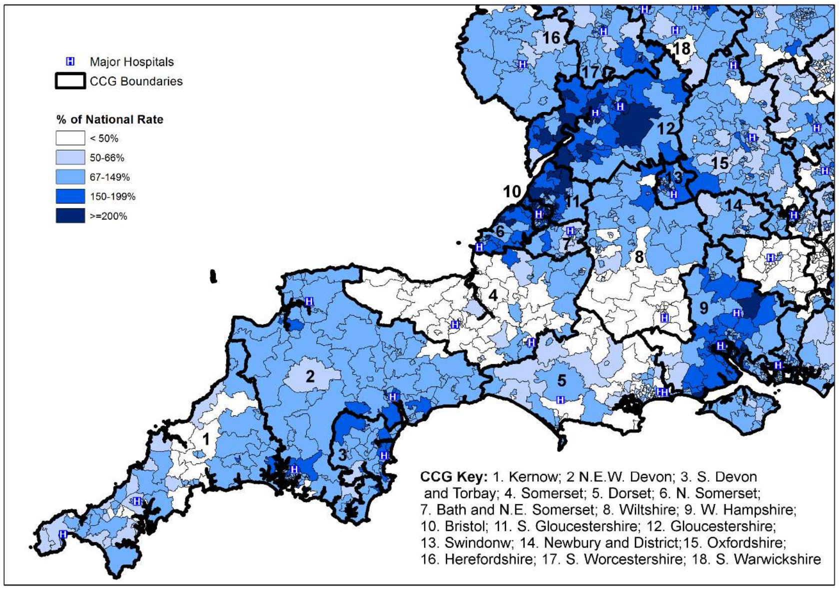

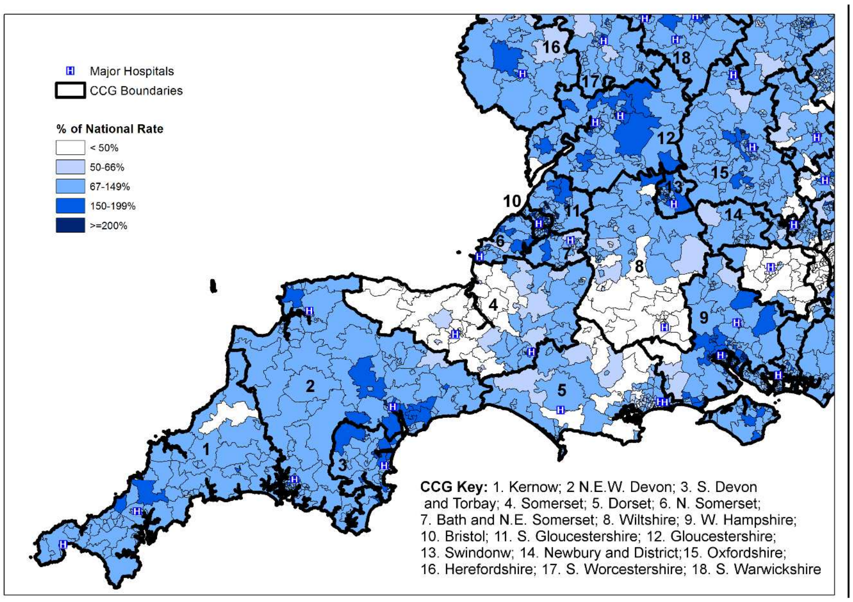

Figure 6a. Indirectly standardised procedure ratios for combined eye injection procedures by MSOA in SW England, 2014/15
Figure 6b. Indirectly standardised patient ratios for combined eye injection procedures by MSOA in SW England, 2014/15
Note: Major hospitals marked including those in NHS Trusts providing 100+ procedures.

296x414mm (300 x 300 DPI)

The RECORD statement – checklist of items, extended from the STROBE statement, that should be reported in observational studies using routinely collected health data.

	Item No.	STROBE items	Location in manuscript where items are reported	RECORD items	Location in manuscript where items are reported
**Title and abstract**					
	1	(a) Indicate the study's design with a commonly used term in the title or the abstract (b) Provide in the abstract an informative and balanced summary of what was done and what was found	P2, L6<br><br>P2, L6-27	RECORD 1.1: The type of data used should be specified in the title or abstract. When possible, the name of the databases used should be included.<br><br>RECORD 1.2: If applicable, the geographic region and timeframe within which the study took place should be reported in the title or abstract.<br><br>RECORD 1.3: If linkage between databases was conducted for the study, this should be clearly stated in the title or abstract.	P2, L6 & 7<br><br>P2, L6-10<br><br>N/A
**Introduction**					
Background rationale	2	Explain the scientific background and rationale for the investigation being reported	P4-5		
Objectives	3	State specific objectives, including any prespecified hypotheses	P5, L21-26		
**Methods**					
Study Design	4	Present key elements of study design early in the paper	P6-9		
Setting	5	Describe the setting, locations, and relevant dates, including periods of recruitment, exposure, follow-up, and data collection	P6, L2-24<br>P9, L4-13		
Participants	6	*(a) Cohort study* - Give the eligibility criteria, and the	P6, L12-24<br>P7, L1-18	RECORD 6.1: The methods of study population selection (such as codes or	P6, L12-24<br>P7, L1-18

For peer review only

		sources and methods of selection of participants. Describe methods of follow-up<br>*Case-control study* - Give the eligibility criteria, and the sources and methods of case ascertainment and control selection. Give the rationale for the choice of cases and controls<br>*Cross-sectional study* - Give the eligibility criteria, and the sources and methods of selection of participants<br><br>*(b) Cohort study* - For matched studies, give matching criteria and number of exposed and unexposed<br>*Case-control study* - For matched studies, give matching criteria and the number of controls per case	N/A	algorithms used to identify subjects) should be listed in detail. If this is not possible, an explanation should be provided.<br><br>RECORD 6.2: Any validation studies of the codes or algorithms used to select the population should be referenced. If validation was conducted for this study and not published elsewhere, detailed methods and results should be provided.<br><br>RECORD 6.3: If the study involved linkage of databases, consider use of a flow diagram or other graphical display to demonstrate the data linkage process, including the number of individuals with linked data at each stage.	P6, L12-17<br><br>N/A
Variables	7	Clearly define all outcomes, exposures, predictors, potential confounders, and effect modifiers. Give diagnostic criteria, if applicable.	P7, L20-24<br>P8, L1-18	RECORD 7.1: A complete list of codes and algorithms used to classify exposures, outcomes, confounders, and effect modifiers should be provided. If these cannot be reported, an explanation should be provided.	P7, L20-24<br>P8, L1-18
Data sources/ measurement	8	For each variable of interest, give sources of data and details of methods of assessment (measurement).<br>Describe comparability of assessment methods if there is more than one group	P7 – P9		
Bias	9	Describe any efforts to address potential sources of bias	P7, L20-24<br>P8, L1-18		
Study size	10	Explain how the study size was	N/A – there was no		

For peer review only

		arrived at	sample size calculation		
Quantitative variables	11	Explain how quantitative variables were handled in the analyses. If applicable, describe which groupings were chosen, and why	P7, L20-24<br>P8, L1-18		
Statistical methods	12	(a) Describe all statistical methods, including those used to control for confounding<br>(b) Describe any methods used to examine subgroups and interactions<br>(c) Explain how missing data were addressed<br>(d) *Cohort study* - If applicable, explain how loss to follow-up was addressed<br>*Case-control study* - If applicable, explain how matching of cases and controls was addressed<br>*Cross-sectional study* - If applicable, describe analytical methods taking account of sampling strategy<br>(e) Describe any sensitivity analyses	P7, L20-24<br>P8, L1-18<br><br>N/A<br><br>N/A<br><br>N/A		
Data access and cleaning methods		..		RECORD 12.1: Authors should describe the extent to which the investigators had access to the database population used to create the study population.<br><br>RECORD 12.2: Authors should provide information on the data cleaning methods used in the study.	P6, L1-11

For peer review only

				P7, L20-24<br>P8, L1-18	
Linkage		..		RECORD 12.3: State whether the study included person-level, institutional-level, or other data linkage across two or more databases. The methods of linkage and methods of linkage quality evaluation should be provided.	N/A
**Results**					
Participants	13	(a) Report the numbers of individuals at each stage of the study (*e.g.*, numbers potentially eligible, examined for eligibility, confirmed eligible, included in the study, completing follow-up, and analysed)<br>(b) Give reasons for non-participation at each stage.<br>(c) Consider use of a flow diagram	Figure 1, P10, L1-7	RECORD 13.1: Describe in detail the selection of the persons included in the study (*i.e.,* study population selection) including filtering based on data quality, data availability and linkage. The selection of included persons can be described in the text and/or by means of the study flow diagram.	Figure 1
Descriptive data	14	(a) Give characteristics of study participants (*e.g.*, demographic, clinical, social) and information on exposures and potential confounders<br>(b) Indicate the number of participants with missing data for each variable of interest<br>(c) *Cohort study* - summarise follow-up time (*e.g.*, average and total amount)	Table 2<br><br>N/A		
Outcome data	15	*Cohort study* - Report numbers of outcome events or summary measures over time<br>*Case-control study* - Report numbers in each exposure category, or summary measures of exposure	Figures 2-6		

For peer review only

		Cross-sectional study - Report numbers of outcome events or summary measures			
Main results	16	(a) Give unadjusted estimates and, if applicable, confounder-adjusted estimates and their precision (e.g., 95% confidence interval). Make clear which confounders were adjusted for and why they were included<br>(b) Report category boundaries when continuous variables were categorized<br>(c) If relevant, consider translating estimates of relative risk into absolute risk for a meaningful time period	Table 2<br>N/A<br>N/A		
Other analyses	17	Report other analyses done—e.g., analyses of subgroups and interactions, and sensitivity analyses	N/A		
**Discussion**					
Key results	18	Summarise key results with reference to study objectives	P12, L1-9		
Limitations	19	Discuss limitations of the study, taking into account sources of potential bias or imprecision. Discuss both direction and magnitude of any potential bias	P12 L11-24<br>P13, L1-4	RECORD 19.1: Discuss the implications of using data that were not created or collected to answer the specific research question(s). Include discussion of misclassification bias, unmeasured confounding, missing data, and changing eligibility over time, as they pertain to the study being reported.	P12 L11-24<br>P13, L1-4
Interpretation	20	Give a cautious overall interpretation of results considering objectives, limitations, multiplicity of analyses, results from similar studies, and other relevant	P15, L20-23<br>P16		

For peer review only

		evidence			
Generalisability	21	Discuss the generalisability (external validity) of the study results	P13, L9-25		
**Other Information**					
Funding	22	Give the source of funding and the role of the funders for the present study and, if applicable, for the original study on which the present article is based	P17		
Accessibility of protocol, raw data, and programming code		..		RECORD 22.1: Authors should provide information on how to access any supplemental information such as the study protocol, raw data, or programming code.	N/A

*Reference: Benchimol EI, Smeeth L, Guttmann A, Harron K, Moher D, Petersen I, Sørensen HT, von Elm E, Langan SM, the RECORD Working Committee. The REporting of studies Conducted using Observational Routinely-collected health Data (RECORD) Statement. *PLoS Medicine* 2015; in press.

*Checklist is protected under Creative Commons Attribution (CC BY) license.

For peer review only

BMJ Open

A longitudinal study to assess the frequency and cost of anti-vascular endothelial therapy, and inequalities in access, in England between 2005 and 2015.

Journal:	*BMJ Open*
Manuscript ID	bmjopen-2017-018289.R2
Article Type:	Research
Date Submitted by the Author:	20-Sep-2017
Complete List of Authors:	Hollingworth, William; University of Bristol, School of Social and Community Medicine<br>Jones, Tim; NIHR CLAHRC West,<br>Reeves, Barnaby; University of Bristol, Bristol Heart Institute<br>Peto, Tunde; Queen's University Belfast Faculty of Medicine Health and Life Sciences
**Primary Subject Heading**:	Health policy
Secondary Subject Heading:	Health economics, Ophthalmology, Evidence based practice, Public health
Keywords:	Vascular Endothelial Growth Factor A, Drug Costs, macular degeneration, macular edema, England, Geography

SCHOLARONE™
Manuscripts

For peer review only

Title: A longitudinal study to assess the frequency and cost of anti-vascular endothelial therapy, and inequalities in access, in England between 2005 and 2015.

Authors: William Hollingworth[1], Tim Jones[2], Barnaby C Reeves[3], Tunde Peto[4]

1 Professor of Health Economics, School of Social and Community Medicine, University of Bristol, Bristol, BS8 2PS, United Kingdom

2 Senior Research Associate, NIHR CLAHRC West, University Hospitals Bristol NHS Foundation Trust, Bristol, BS1 2NT United Kingdom

3 Professorial Research Fellow in Health Services Research, Clinical Trials Evaluation Unit, School of Clinical Sciences, University of Bristol, Bristol, BS2 8HW, United Kingdom

4 Professor of Clinical Ophthalmology, School of Medicine, Dentistry and Biomedical Sciences, Queen's University Belfast, Belfast, BT9 7BL, United Kingdom

Corresponding author:

William Hollingworth
Professor of Health Economics
Health Economics at Bristol (HEB)
School of Social and Community Medicine
University of Bristol
Canynge Hall
39 Whatley Road
Bristol, BS8 2PS
United Kingdom

Tel: ++44 (0)117 9287355
william.hollingworth@bristol.ac.uk

Word count [3,564]

Keywords:
Vascular Endothelial Growth Factor A; Drug Costs; macular degeneration; macular edema; England; socioeconomic factors; geography

For peer review only

Abstract [289 words]

Objectives: High cost anti-VEGF medicines for eye disorders challenge ophthalmologists and policymakers to provide fair access for patients while minimising costs. We describe the growth in the use and costs of these medicines and measure inequalities in access.

Design: Longitudinal study using hospital episode statistics (2005/6 to 2014/15) and hospital prescribing cost reports (2008/9 to 2015/16). We used Poisson regression to estimate standardised rates and explore temporal and geographic variations.

Setting: NHS care in England.

Population: Patients receiving anti-VEGF injections for age-related macular degeneration, diabetic macular oedema and other eye disorders.

Interventions: Higher cost drugs (ranibizumab or aflibercept) recommended by NICE or lower cost drug (bevacizumab) not licensed for eye disorders.

Main outcome measures: National procedure rates and variation between and within clinical commissioning groups (CCGs). Cost of ranibizumab and aflibercept prescribing.

Results: Injection procedures increased by 215% between 2010/11 and 2014/15. In 2014/15 there were 388,031 procedures (714 per 100,000). There is no evidence that the dramatic growth in rates is slowing down. Since 2010/11 the estimated cost of ranibizumab and aflibercept increased by 247% to £447 million in 2015/16, equivalent to the entire annual budget of a CCG. There are large inequalities in access; in 2014/15 procedure rates in a 'high use' CCG were 9.08 times higher than in a 'low use' CCG. In the South West of England there was twofold variation in injections per patient per year (range 2.9 to 5.9).

Conclusions: The high and rising cost of anti-VEGF therapy affects the ability of the NHS to provide care for other patients. Current regulations encourage the increasing use of ranibizumab and aflibercept rather than bevacizumab, which evidence suggests is more cost-effective. NHS patients in England do not have equal access to the most cost-effective care.

For peer review only - http://bmjopen.bmj.com/site/about/guidelines.xhtml

Strengths and limitations

- Our study summarises new data on nationwide access to effective, but high cost ophthalmology drugs.
- Using population denominators allows us to standardise our analysis for factors, such as age and socio-economic deprivation, which will affect the need for care.
- The lack of a specific anti-VEGF drug code in HES is a limitation, however we observed similar temporal trends in two independent data sources.
- The confidential nature of drug discounts mean that our estimate of drug costs will be too high if commissioners have negotiated substantial discounts.
- Conversely, the exclusion of administration and monitoring costs make our estimates conservative.

For peer review only

For peer review only - http://bmjopen.bmj.com/site/about/guidelines.xhtml

Introduction

Clinical commissioning groups (CCGs) in England are responsible for commissioning healthcare for a geographically defined local population. On average, CCGs cover a population of 270,000 and have a budget of £300 million to commission services including elective and unplanned hospital care, community and mental health services. While CCGs have a degree of autonomy in the care they commission, they must work within national constraints. In particular, CCGs must commission new interventions which have been evaluated to be cost-effective by the National Institute for Health and Care Excellence (NICE) technology appraisal process. This presents a challenge as many of these interventions are expensive. A balance is needed in order to provide fair access for patients eligible for the new interventions without disadvantaging other patients using existing health services.

One recent challenge for CCGs is to commission anti vascular endothelial growth factor (anti-VEGF) medicines for patients with a range of eye conditions. Anti-VEGF intravitreal injections reduce new blood vessel growth and swelling [1]. Two drugs (ranibizumab and aflibercept) are licensed for use in neovascular age-related macular degeneration (nAMD), macular oedema due to diabetes (DMO) or retinal vein occlusion (RVO), and myopic choroidal neovascularisation (MCNV). Ranibizumab was first recommended by NICE as cost-effective for use in nAMD in 2008 [2]. After an initial rejection in 2011, NICE also recommended the use of ranibizumab for DMO in 2013 based on new evidence and an undisclosed discount on price offered by the manufacturer [3]. Subsequent NICE recommendations in other eye conditions and for aflibercept have followed (Table 1). Ranibizumab and aflibercept have an NHS indicative price in the region of £550 and £800 per injection, respectively [4]. The high prevalence of these conditions, the indefinite need for multiple injections per patient, and the associated procedure and monitoring costs mean that economic burden is substantial.

In reaching their recommendations, NICE did not compare ranibizumab or aflibercept with a pre-existing drug, bevacizumab. Bevacizumab also inhibits VEGF and is closely related to ranibizumab; it

For peer review only - http://bmjopen.bmj.com/site/about/guidelines.xhtml

is licensed as a treatment for certain types of cancer but not in ophthalmology. Bevacizumab can be divided into aliquots of smaller doses for ophthalmic therapy and is a much less costly alternative (approximately £50 to £100 per injection) [5]. Meta-analysis has demonstrated that bevacizumab and ranibizumab result in similar visual acuity outcomes for patients with nAMD [6]. The CATT and IVAN randomised trials of ranibizumab versus bevacizumab in nAMD both demonstrated that the drugs resulted in similar improvements in visual acuity [7, 8]. IVAN also found that ranibizumab produced little or no gain in quality-adjusted life years (QALYs) despite being substantially more expensive [9]. Individual patient data meta-analysis indicates that the risk of serious adverse events with ranibizumab and bevacizumab are similar in nAMD [10]. In DMO, the DRCR.net three-arm randomised trial found that aflibercept and ranibizumab were not cost-effective relative to bevacizumab despite evidence that aflibercept resulted in better visual outcomes at 1 and 2 years, particularly in those with worse visual acuity at baseline [11, 12]. In RVO, the SCORE2 randomised trial concluded that bevacizumab was non-inferior to aflibercept for visual acuity after 6 months of treatment [13]. In 2014, the Royal College of Ophthalmologists and others called on UK health regulators, including NICE, to urgently review the use of bevacizumab in nAMD [14]. However, in 2015 the Department of Health declined to support clinicians or commissioners using bevacizumab for nAMD, considering unlicensed use "on ground of cost alone" as potentially illegal under EU law [15], despite political support for the use of bevacizumab for ophthalmic indications in other EU countries such as Italy [16]. The remit of the forthcoming NICE nAMD clinical guidelines preclude making recommendations about bevacizumab [17].

The emergence of anti-VEGF medicines provides ophthalmologists and CCGs with a clinical and economic dilemma in both providing fair access for eligible patients to drugs recommended by NICE while minimising the opportunity costs for other members of the population. In this study, we describe the growth in the use and cost of anti-VEGF intravitreal injections in the English NHS since 2005/06. We quantify the variation in patient access to anti-VEGF therapy across England and explore the potential causes of inequality of access within the South West region of England.

For peer review only - http://bmjopen.bmj.com/site/about/guidelines.xhtml

Methods

IDENTIFYING ANTI-VEGF INJECTIONS

We used the HES admitted patient care (APC) and outpatient (OP) datasets to identify anti-VEGF injections. Anti-VEGF injections are performed in outpatient treatment rooms, but, in earlier years, were often coded in the APC dataset as day case procedures. HES is a routinely collected dataset that records all episodes of care provided to NHS funded and privately insured patients admitted (day case or inpatient) to NHS hospitals or seen at an NHS outpatient clinic [18]. HES also includes data on NHS-funded patients treated in independent sector hospitals. We extracted anonymised individual episode records on all episodes contained in the HES APC (2005/6 to 2014/15) and OP (2010/11 to 2014/15) datasets. Up to 24 clinical procedures per episode may be recorded using Office of Population, Censuses and Surveys (OPCS) (fourth revision) codes.

Anti-VEGF injection procedures are not coded consistently. The majority of healthcare providers used the code 'injection into vitreous body, not elsewhere classified [C79.4]'. In preliminary exploration of HES data we looked for negative correlations between this procedure code and other ophthalmology procedure codes to identify potential alternate procedure codes. This identified that a substantial minority used a newer code 'Injection of therapeutic substance into posterior segment of eye, not elsewhere classified [C89.3]'. HES does not record the specific drug injected into the eye; however, since 2009 an OPCS code for high cost subfoveal choroidal neovascularisation drugs Band 1 [X93.1] has been in use. This code may be used for aflibercept, bevacizumab, ranibizumab, and two other drugs rarely used in intravitreal injections, pegaptanib and verteporfin. We excluded any procedures using non anti-VEGF drugs (X93.2, X93.3).

In 2014/15, 73.3% (284,490/388,031) of episodes including an eye injection procedure also coded a high cost subfoveal choroidal neovascularisation drug. However, some hospitals performing hundreds of intravitreal injections each year never use this high cost drug code. Conversely, 96%

For peer review only - http://bmjopen.bmj.com/site/about/guidelines.xhtml

(284,490/296,129) of episodes coding a high cost subfoveal choroidal neovascularisation drug also included an eye injection procedure (Figure 1). In our analysis, we assumed that procedures coded as C79.4 or C89.3 were anti-VEGF injections. We explore the importance of this assumption in our discussion.

A minority of patients receive bilateral injections on the same day, which may be coded using the supplemental 'bilateral operation' [Z94.1] OPCS code. When a patient had a bilateral procedure coded, we counted this as two injection procedures. When a patient had two injection procedures coded on the same day without any supplemental bilateral code, we assumed that this also represented bilateral procedures. If patients had more than two injection procedures coded on the same day, we excluded the 'excess' procedures as probable coding error duplicates (<0.03% of all procedures).

We used ICD-10 diagnosis codes to identify patients with diabetes with ophthalmic complications (E10.3, E11.3, E12.3, E13.3, or E14.3) or diabetic retinopathy (H36.0) or with a primary code indicating “other specified retinal disorders” (H35.8) combined with secondary codes indicating diabetes with ophthalmic complications as above; nAMD (H35.3); RVO (H34.8); and MCNV (H44.2) respectively. Diagnosis codes are well recorded for procedures in the HES APC dataset, however in the HES OP dataset diagnosis codes are often missing. Therefore, we limited our analysis of diagnostic indications to procedures recorded in the HES APC dataset.

ESTIMATING PROCEDURE RATES

National trends over time were estimated using directly standardised procedure rates [19] (per 100,000 population), with the population of England in 2014 as our standard population. We first summed the number of anti-VEGF injections, grouped by sex, quintiles of age, and financial year. These procedure counts were used to calculate annual age-sex-specific rates, by dividing by the appropriate age-sex-specific mid-year populations of England [20] (e.g. for the 2012/13 financial

For peer review only - http://bmjopen.bmj.com/site/about/guidelines.xhtml

year, the mid-2012 populations were used). We weighted the annual age-sex-specific rates according to the population distribution of England in 2014, to produce directly standardised rates for each year.

Patients typically have more than one injection procedure in a year. Variation in patient access to anti-VEGF injections within England might be evident in both the number of patients treated and the number of procedures performed. Therefore, we estimated indirectly standardised rates [19] per 100,000 population for both patients and procedures. We first calculated age-sex-specific rates for England in 2014/15, then multiplied these rates by the age-sex-specific population for the area of interest [20-22] (e.g. CCG) and summed the results. This produced the expected number of patients and procedures for that area, if it were to have the same age-sex-specific rates as England. The expected number was then compared to the observed number of patients and procedures for that area. A Poisson regression model was fitted to the observed counts, with the expected counts as an offset and socio-economic deprivation (using the overall score from the English Indices of Multiple Deprivation[23]) and ethnicity (% white British[24]) as predictive factors. The model was then used to predict new expected counts for each area based on deprivation and ethnicity, and form indirectly standardised patient and procedure ratios (observed / expected). Statistical analyses were conducted using Stata/MP 14.2 for Windows and we mapped variation in patient and procedure rates across England in 2014/15 using ArcGIS ArcMap 10.3.1 for Desktop.

EXPLORING VARIATION IN ACCESS IN THE SOUTH WEST REGION OF ENGLAND

For each CCG in the South West region of England we estimated the median age and gender proportion in order to explore any differences in the demographic profile of patients receiving anti-VEGF injections. We estimated the annual number of procedures per patient in 2014/15 to determine whether CCGs with high patient rates also had a higher number of procedures per patient. We also calculated and mapped indirectly standardised procedure ratios between Middle

For peer review only - http://bmjopen.bmj.com/site/about/guidelines.xhtml

Layer Super Output Areas (MSOA) to explore whether CCG boundaries or hospital catchment areas were more strongly associated with access to anti-VEGF injections.

ESTIMATING THE COST OF ANTI-VEGF INJECTIONS

We extracted data from NHS Digital Hospital prescribing costs reports [25] in order to estimate the costs of ranibizumab and aflibercept since 2008/9 when ranibizumab was first approved by NICE. The NHS Digital report summarises data collected from English NHS hospital pharmacies by the IMS Health Hospital Pharmacy Audit Index (HPAI). This includes high cost medicines supplied to wards, outpatient clinics and satellite sites. Costs are reported in the HPAI and are calculated by IMS Health using standard price lists and therefore do not necessarily reflect any discounted prices paid by hospitals. Anti-VEGF therapy also incurs administration and monitoring costs; however, the IVAN trial indicated that, in the ranibizumab group, drug costs accounted for 80-88% of total costs [9]. Injection frequency and monitoring visits may be lower for aflibercept where fixed 2-monthly interval dosing is recommended after an initial period of monthly loading doses.

Bevacizumab is not included in the HPAI and due to the lack of drug coding in HES data it is impossible to accurately estimate the current use of bevacizumab for eye conditions. A study in 2015 based on 189 Freedom of Information requests across the UK estimated the relative prevalence of injections of ranibizumab, aflibercept and bevacizumab to be 61.1%, 36.0% and 2.8% respectively [26].

For peer review only - http://bmjopen.bmj.com/site/about/guidelines.xhtml

Results

THE USE AND COST OF ANTI-VEGF INTRAVITREAL INJECTIONS

In 2014/15 there were 388,031 intra-vitreal injection procedures, equivalent to 714 procedures per 100,000 population (Figure 2). This compares to 123,006 procedures in 2010/11 and represents a 215% increase in the number of procedures over the five-year period. Before 2007, intra-vitreal injections were rarely used. The rise in procedure rates since then is dramatic and there are no signs that it is levelling off.

Amongst episodes recorded in the HES APC dataset, the majority (62% in 2014/15) had diagnosis codes indicating nAMD (Figure 3). The relative use of diagnosis codes indicating DMO or RVO show small recent increases associated with NICE approvals for these indications in 2013. The estimated total cost of hospital prescribing of ranibizumab and aflibercept increased by 197% from £129 million to £383 million in the five years between 2010/11 and 2014/15 (Figure 4). The estimated total cost of ranibizumab and aflibercept had risen to £447 million in 2015/16, predominantly due to the increasing use of aflibercept.

VARIATION IN PATIENT ACCESS TO ANTI-VEGF THERAPY

In 2014/15 there was substantial variation in procedure rates between CCGs, even after adjusting for age, sex, deprivation, and ethnicity profiles (Figure 5a). The map demonstrates pockets of very high use (>150% times the expected rate), for example in the Bristol area and in parts of North London and Norfolk. There are also areas where procedure rates are less than 50% of the expected rate, for example in parts of Essex. There was less pronounced, but still substantial variation in patient rates between CCGs, after adjusting for age, sex, deprivation and ethnicity profiles (Figure 5b). Areas of the country with high procedure rates also tended to have higher proportions of their populations having treatment. In 2014/15 the ratio in patient rates between a 'high use' CCG at the 90th percentile and a 'low use' CCG at the 10th percentile was 4.98. This ratio has only slightly decreased

For peer review only

For peer review only - http://bmjopen.bmj.com/site/about/guidelines.xhtml

since 2010/11, when the ratio was 6.07, indicating that equality of access to care is only slowly improving over time.

Examining the South West region of England in more detail (Figures 6a and 6b), it is evident that there is substantial variation in access to anti-VEGF injections within CCGs. For example, in both Somerset and Wiltshire there are areas of relatively high procedure rates closer to the major conurbations of Bath, Bristol and Swindon. Procedure rates are much lower than expected in other areas of these CCGs, even though these other areas are served by local hospitals. The age and gender profile of patients receiving anti-VEGF therapy was similar between CCGs (Table 2). Median age ranged from 77 to 82 years and between 39% and 49% of patients were male. There was twofold variation in the number of procedures per patient per year ranging from 2.9 in Cornwall to 5.9 in South Gloucestershire. The standardised CCG patient rate was strongly correlated with the number of procedures per patient (Spearman's Rho = 0.71, $p < .001$).

For peer review only - http://bmjopen.bmj.com/site/about/guidelines.xhtml

Discussion

STATEMENT OF PRINCIPAL FINDINGS

There is no evidence that the dramatic growth in the use of high cost anti-VEGF injections in ophthalmology is slowing down. Injection procedures increased by 215% between 2010/11 and 2014/15. There are large inequalities in access to anti-VEGF therapy. This inequality is not explained by differences in demographics and occurs both within and between CCGs. Procurement of high cost anti-VEGF drugs has placed a large and increasing strain on the NHS. In 2015/16, the NHS spent an estimated £447 million on drug costs alone, equivalent to the entire annual budget of a large CCG.

STRENGTHS AND WEAKNESSES OF THE STUDY

Our study summarises new data on nationwide access to effective, but high cost ophthalmology drugs. Using population denominators allows us to standardise our analysis for factors, such as age and socio-economic deprivation, which will affect the need for care. HES data records patients' area of residence, so we compare access based on place of residence rather than place of treatment. The combination of HES APC and OP datasets is essential in order to accurately monitor intravitreal injections over time. As reimbursement payments depend on procedure coding, there is an incentive for providers to fully code procedures. However, the lack of a specific anti-VEGF drug code in HES is a limitation. Other similar procedures such as the intravitreal injection of dexamethasone implants have distinct OPCS procedure codes, although we cannot exclude the possibility of miscoding. We observed similar temporal trends in two independent data sources measuring procedures (i.e. HES) and drug costs (i.e. HPAI) which provides reassurance about the validity of our time-trend analyses. Previous work has demonstrated that HES generally has good sensitivity in identifying new procedures, but may lack specificity particularly in the absence of a single specific procedure code [27]. It is plausible that drug price discounts account for some of the observed

variations, if CCGs who have larger discounts offer more therapy. The confidential nature of discounts mean that our estimate of drug costs will be too high if CCGs have negotiated substantial discounts. Conversely, the exclusion of administration and monitoring costs make our estimates conservative and there is a substantial economic burden for patients. Travel and post-appointment recovery time have a large impact on patients and carers [28]. The costs of all three anti-VEGF drugs will be offset, to a greater or lesser extent, in the longer term by reduced need for the NHS and social services related to vision loss.

COMPARISON WITH OTHER STUDIES

Between 1989/90 and 2008/9, Keenan et al observed an increase in intravitreal injection rates from 0.4 per 100,000 to 59.5 per 100.000 with wide variations in access between different local authorities in England [29]. Our data demonstrate that this rapid growth has continued and even accelerated in the following six years. We were also able to explore variation in access to care at a more granular level and demonstrate that access varies markedly both within and between CCGs, suggesting that variations are likely to be due, at least in part, to the availability of trained staff (e.g. ophthalmologists and nurse practitioners) and facilities in local hospitals rather than CCG-wide policies. Using insurance data, Parikh et al observed a continually increasing trend in anti-VEGF use in the US between 2006 and 2015 [30]. In line with our study, Parikh et al also found that anti-VEGF drug use was predominantly for nAMD. It is impossible to directly compare England and US rates as the denominator used in the Parikh et al study excludes patients without commercial or Medicare Advantage insurance. However, anti-VEGF therapy rates in the US and UK appear to be of the same order of magnitude. Specific coding of anti-VEGF drugs allowed Parikh et al to document the rise in the use of aflibercept (28% of all anti-VEGF injections in 2015) and the concurrent levelling off in the use of ranibizumab (17%) and bevacizumab (55%). This is in stark contrast to England where ranibizumab and aflibercept predominate. This is likely to be a result of legal challenges to the use of bevacizumab and the lack of Department of Health support for commissioners [31].

For peer review only - http://bmjopen.bmj.com/site/about/guidelines.xhtml

IMPLICATIONS FOR POLICYMAKERS AND CLINICIANS

Based on the IVAN trial, Dakin et al estimated that the NHS could save £102 million per year by switching from ranibizumab to bevacizumab [9]. In the US, Hutton et al estimated that a similar switch would save Medicare $18 billion over a 10-year period [32]. Given the lack of political and regulatory support for clinicians to use bevacizumab, it is unsurprising that most in England do not use it. This has led to the very unusual situation whereby the NHS is paying for more expensive therapy than healthcare insurers in the US. The patent on ranibizumab is due to expire in Europe in 2022 [33]; in the meantime, based on current trends, the NHS may spend billions on anti-VEGF injections, before biosimilar drugs become available. Our data, demonstrating large variations between CCGs in the numbers of patients accessing anti-VEGF services also suggests that there is considerable potential unmet need in some areas of the country. Given that many CCGs are already in financial deficit, most will struggle to afford to treat more patients unless they are able to switch to bevacizumab. The political decision not to support NHS use of bevacizumab in eye conditions is in stark contrast to decisions taken in other EU countries [16] and has very large negative consequences for NHS patients.

In April 2017, NICE introduced a budget impact test for new technologies under appraisal that are expected to cost more than £20 million per annum. This allows NHS England up to three years to conduct commercial negotiations on price rather than the current requirement to fund within 90 days [34]. This policy should strengthen the future negotiating power of the NHS for expensive drugs. However, it does not address the fundamental problem that NICE has not included cost-effective but unlicensed drugs in technology appraisals or clinical guideline recommendations.

The widespread variation in procedure rates that we observed might be due to travel distance affecting patient access to diagnostic and therapeutic services, particularly in more remote areas. Lengthy travel distance is associated with treatment discontinuance [35]. However, many of the low injection rate areas in the South West of England are close to hospitals. Other unmeasured factors,

For peer review only - http://bmjopen.bmj.com/site/about/guidelines.xhtml

such as the importance of vision for work or usual activities, may also be associated with patient uptake and compliance with anti VEGF therapy. The frequency of injections per patient per year was lower than reported in trials of therapy initiation. The averages in our study will be drawn downwards due to patients starting and discontinuing therapy partway through the year and by patients who have been on anti-VEGF therapy for many years and need fewer injections.

UNANSWERED QUESTIONS AND FUTURE RESEARCH

Given the high cost of the drugs, it is imperative that clinical services are organised to deliver care efficiently. For example, nurse-administered injections [36], treat and extend protocols [37], optometrist-led monitoring [38] and stereotactic radiotherapy [39] all offer the potential to reduce costs and require stronger evidence. The wide variation between CCGs in the number of injections per patient represents a natural experiment. Observational studies could compare costs and outcomes between areas of the country with high and low injection frequency. However, the lack of a nationwide register of patients receiving anti-VEGF injections and the lack of drug coding in hospital episode statistics severely limit the potential to conduct this research.

CONCLUSIONS

Anti-VEGF therapy is effective in most cases and has improved the quality of life of many thousands of patients in England. However, current regulations encourage the increasing use of ranibizumab and aflibercept rather than bevacizumab, which evidence suggests is more cost-effective. This limits the ability of the NHS to pay for care for other patients. NHS patients in England do not have equal access to the most cost-effective care.

For peer review only - http://bmjopen.bmj.com/site/about/guidelines.xhtml

Acknowledgements:
Hospital Episode Statistics were provided by NHS Digital under data sharing agreement (NIC-17875-X7K1V) with the University of Bristol. Copyright © 2016, re-used with the permission of The Health & Social Care Information Centre. All rights reserved.

Funding:
William Hollingworth and Tim Jones receive funding from the National Institute for Health Research Collaboration for Leadership in Applied Health Research and Care (NIHR CLAHRC) West. The views expressed are those of the authors and not necessarily those of the NHS, the NIHR or the Department of Health. The funder had no role in the study design, collection, analysis and interpretation of data or in the writing of the report and the decision to submit the article for publication.

Competing Interests:
All authors have completed the Unified Competing Interest form (available on request from the corresponding author) and declare: no support from any organisation for the submitted work; no financial relationships with any organisations that might have an interest in the submitted work in the previous three years, no other relationships or activities that could appear to have influenced the submitted work.

Copyright statement:
The Corresponding Author has the right to grant on behalf of all authors and does grant on behalf of all authors, a worldwide licence to the Publishers and its licensees in perpetuity, in all forms, formats and media (whether known now or created in the future), to i) publish, reproduce, distribute, display and store the Contribution, ii) translate the Contribution into other languages, create adaptations, reprints, include within collections and create summaries, extracts and/or, abstracts of the Contribution, iii) create any other derivative work(s) based on the Contribution, iv) to exploit all subsidiary rights in the Contribution, v) the inclusion of electronic links from the Contribution to third party material where-ever it may be located; and, vi) licence any third party to do any or all of the above.

We attest that we have obtained appropriate permissions and paid any required fees for use of copyright protected materials.

Transparency declaration:
The lead author affirms that the manuscript is an honest, accurate, and transparent account of the study being reported; that no important aspects of the study have been omitted; and that any discrepancies from the study as planned (and, if relevant, registered) have been explained.

Details of contributors:
WH initiated and designed the study, contributed to the data analysis and interpretation of data and drafted and revised the paper. He is guarantor. TJ contributed to the study design, cleaned and analysed the data, contributed to the interpretation of data and revised the draft paper. BR and TP contributed to the interpretation of data and revised the draft paper. All authors approved the final version of the paper. All authors had full access to all of the data in the study and can take responsibility for the integrity of the data and the accuracy of the data analysis.

Ethical approval:

For peer review only - http://bmjopen.bmj.com/site/about/guidelines.xhtml

The paper describes work based on anonymised individual episode records provided under a data sharing agreement between NHS Digital and the University of Bristol. The data were not linked to other datasets or re-identified. Ethical approval was not required.

Data sharing:
No additional data available.

For peer review only

For peer review only - http://bmjopen.bmj.com/site/about/guidelines.xhtml

Figures

Figure 1 – OPCS codes used to identify anti-VEGF injections: 2014/15

Note: 2,479 eye injection procedures were excluded due to combination with X93.2 or X93.3 high-cost drug codes.

Figure 2. Directly-standardised intravitreal injection procedure rates, England, 2005/06-2014/15

Note: OP = Outpatient; APC = Admitted Patient Care

Figure 3. Percentage of total eye injection procedures in HES APC for different primary diagnoses, England, 2008/09-2014/15.

Note: nAMD = Neovascular Age-Related Macular Degeneration; DMO = Diabetic Macular Oedema; RVO = Retinal Vein Occlusion; Due to very small numbers of procedures for Myopic Choroidal Neo-Vascularisation (<=10 per year), this was included in 'Other'.

Figure 4. Hospital prescribing costs for Ranibizumab and Aflibercept, 2008/09 to 2015/16

Figure 5a. Indirectly age-sex-deprivation-ethnicity standardised procedure ratios for combined eye injection procedures (OPCS-4: C794 and C893) by CCG in England, 2014/15.

Figure 5b. Indirectly age-sex-deprivation-ethnicity standardised patient ratios for combined eye injection procedures (OPCS-4: C794 and C893) by CCG in England, 2014/15.

Figure 6a. Indirectly standardised procedure ratios for combined eye injection procedures by MSOA in SW England, 2014/15

Note: Major hospitals marked including those in NHS Trusts providing 100+ procedures.

Figure 6b. Indirectly standardised patient ratios for combined eye injection procedures by MSOA in SW England, 2014/15

Note: Major hospitals marked including those in NHS Trusts providing 100+ procedures.

For peer review only

Table 1 – NICE appraisals for anti-VEGF therapies

Condition	Ranibizumab	Aflibercept
nAMD	Recommend 8/2008 [TA 155]	Recommend 7/2013 [TA 294]
DMO	Reject 11/2011 [TA 237]<br>Recommend 2/2013 [TA 274]	Recommend 7/2015 [TA 346]
RVO	Recommend 5/2013 [TA 294]	Recommend 2/2014 [TA 305]
MCNV	Recommend 11/2013 [TA 298]	Recommend 9/2016 [TA 409]

For peer review only

For peer review only - http://bmjopen.bmj.com/site/about/guidelines.xhtml

Table 2. Demographics of anti-VEGF procedures in South West CCGs, 2014/15; ordered by procedures per patient.

CCG Name	% Male	Median Age	Procedures per Patient (95% CI)	Procedure Rate* (95% CI**)	Patient Rate* (95% CI)
NHS Kernow CCG	43	79	2.86 (2.75-2.97)	522 (490-556)	172 (191-104)
NHS Dorset CCG	43	82	3.32 (3.21-3.43)	419 (394-445)	122 (136-74)
NHS Herefordshire CCG	38	80	3.34 (3.14-3.53)	631 (569-700)	175 (210-110)
NHS Somerset CCG	45	78	3.48 (3.27-3.68)	304 (276-334)	92 (106-57)
NHS South Warwickshire CCG	44	79	3.63 (3.46-3.8)	773 (708-844)	206 (240-128)
NHS Oxfordshire CCG	40	81	3.63 (3.5-3.76)	723 (679-771)	197 (220-120)
NHS Northern Eastern and Western Devon CCG	41	81	4.05 (3.95-4.16)	830 (792-869)	201 (218-121)
NHS Newbury and District CCG	41	79	4.14 (3.72-4.55)	637 (530-764)	136 (186-92)
NHS Wiltshire CCG	46	79	4.17 (3.95-4.39)	459 (419-502)	103 (121-64)
NHS South Devon and Torbay CCG	40	81	4.2 (4.05-4.35)	923 (860-991)	206 (235-127)
NHS South Worcestershire CCG	42	79	4.36 (4.16-4.56)	875 (806-950)	188 (217-116)
NHS Bath and North East Somerset CCG	49	81	4.44 (4.14-4.73)	735 (650-831)	152 (189-97)
NHS Swindon CCG	44	77	4.54 (4.26-4.82)	1034 (928-1153)	216 (260-137)
NHS West Hampshire CCG	42	80	5 (4.85-5.15)	1125 (1063-1190)	221 (244-133)
NHS North Somerset CCG	43	80	5.43 (5.15-5.71)	1236 (1130-1352)	217 (253-135)
NHS Bristol CCG	43	80	5.49 (5.26-5.72)	1346 (1248-1451)	237 (270-146)
NHS Gloucestershire CCG	42	81	5.51 (5.33-5.68)	1263 (1194-1336)	226 (249-137)
NHS South Gloucestershire CCG	46	79	5.93 (5.65-6.22)	1379 (1264-1505)	222 (258-138)

** Procedure rate and patient rate are the indirectly standardised rates per 100,000 population*

*** 95% CIs on the procedure rate are calculated using Stukel et al.'s method for recurrent outcomes[40]*

For peer review only - http://bmjopen.bmj.com/site/about/guidelines.xhtml

References

1. Steinbrook, R., *The price of sight--ranibizumab, bevacizumab, and the treatment of macular degeneration.* N Engl J Med, 2006. **355**(14): p. 1409-12.
2. National Institute for Health and Care Excellence, *Ranibizumab and pegaptanib for the treatment of age-related macular degeneration*. 2008.
3. National Institute for Health and Care Excellence, *Ranibizumab for treating diabetic macular oedema*. 2013.
4. BMJ Group and Pharmaceutical Press, *British National Formulary*. 2017: London.
5. Poku, E., et al., *Bevacizumab in eye conditions: Issues related to quality, use, efficiency and safety*. 2012, NICE Decision Support Unit: Sheffield.
6. Solomon, S.D., et al., *Intravitreal Bevacizumab Versus Ranibizumab for Treatment of Neovascular Age-Related Macular Degeneration: Findings from a Cochrane Systematic Review.* Ophthalmology, 2016. **123**(1): p. 70-77 e1.
7. Chakravarthy, U., et al., *Alternative treatments to inhibit VEGF in age-related choroidal neovascularisation: 2-year findings of the IVAN randomised controlled trial.* Lancet, 2013. **382**(9900): p. 1258-67.
8. Comparison of Age-related Macular Degeneration Treatments Trials Research, G., et al., *Ranibizumab and bevacizumab for treatment of neovascular age-related macular degeneration: two-year results.* Ophthalmology, 2012. **119**(7): p. 1388-98.
9. Dakin, H.A., et al., *Cost-effectiveness of ranibizumab and bevacizumab for age-related macular degeneration: 2-year findings from the IVAN randomised trial.* BMJ Open, 2014. **4**(7): p. e005094.
10. Maguire, M., et al., *Serious Adverse Events with Bevacizumab or Ranibizumab for Age-Related Macular Degeneration: Meta-analysis of Individual Patient Data.* Ophthalmology Retina, In Press.
11. Ross, E.L., et al., *Cost-effectiveness of Aflibercept, Bevacizumab, and Ranibizumab for Diabetic Macular Edema Treatment: Analysis From the Diabetic Retinopathy Clinical Research Network Comparative Effectiveness Trial.* JAMA Ophthalmol, 2016. **134**(8): p. 888-96.
12. Wells, J.A., et al., *Aflibercept, Bevacizumab, or Ranibizumab for Diabetic Macular Edema: Two-Year Results from a Comparative Effectiveness Randomized Clinical Trial.* Ophthalmology, 2016. **123**(6): p. 1351-9.
13. Scott, I.U., et al., *Effect of Bevacizumab vs Aflibercept on Visual Acuity Among Patients With Macular Edema Due to Central Retinal Vein Occlusion: The SCORE2 Randomized Clinical Trial.* JAMA, 2017. **317**(20): p. 2072-2087.
14. Lotery, A. and C. MacEwen, *What is stopping the NHS from using bevacizumab for macular degeneration and other retinal disorders?* BMJ, 2014. **349**: p. g6887.
15. Freeman, G., *Letter to NHS Southern Derbyshire CCG*, Department of Health, Editor. 2015.
16. Formoso, G., et al., *A clearer view of evidence in treating macular degeneration: off-label policies and independent research.* Cochrane Database Syst Rev, 2014(9): p. ED000090.
17. National Institute for Health and Care Excellence, *Guideline scope age-related macular degeneration: diagnosis and management* 2015, NICE.
18. Health and Social Care Information Centre, *Hospital Episode Statistics (HES) Analysis Guide*. 2015.
19. Anon, *Standardization: a classic epidemiological method for the comparison of rates.* Epidemiological Bulletin, 2002. **23**(3): p. 9-12.
20. Office for National Statistics. *Lower Super Output Area Mid-Year Population Estimates* [cited 2016; Available from: https://www.ons.gov.uk/peoplepopulationandcommunity/populationandmigration/populationestimates/datasets/lowersuperoutputareamidyearpopulationestimates.

For peer review only - http://bmjopen.bmj.com/site/about/guidelines.xhtml

21. Office for National Statistics. *Lower Layer Super Output Area (2011) to Clinical Commissioning Group to Local Authority District (July 2015) Lookup in England*. [cited 2016; Available from: http://ons.maps.arcgis.com/home/item.html?id=7de88cd2369d4006925de49e8f3f2e27.
22. Office for National Statistics. *Output Area to Lower Layer Super Output Area to Middle Layer Super Output Area to Local Authority District (December 2011) Lookup in England and Wales*. [cited 2016; Available from: http://ons.maps.arcgis.com/home/item.html?id=baf6566ca08949c6bbd61ff81d9526da.
23. Government, D.o.C.a.L. *English Indices of Deprivation 2015*. [cited 2016; Available from: https://www.gov.uk/government/statistics/english-indices-of-deprivation-2015.
24. Nomis; Office for National Statistics. *Ethnic group of the usual resident population of England and Wales as at census day, 27 March 2011*. [cited 2016; Available from: https://www.nomisweb.co.uk/census/2011/ks201ew.
25. NHS Digital, *Prescribing Costs in Hospitals and the Community*. 2016.
26. Di Simplicio, S., et al. *A UK survey of anti-VEGF use for the eye in January 2015*. in *The Association for Research in Vision and Ophthalmology Annual Meeting*. 2017. Baltimore, MD.
27. Patrick, H., et al., *Monitoring the use and outcomes of new devices and procedures: how does coding affect what Hospital Episode Statistics contribute? Lessons from 12 emerging procedures 2006-10.* J Public Health (Oxf), 2013. **35**(1): p. 132-8.
28. Prenner, J.L., et al., *Disease Burden in the Treatment of Age-Related Macular Degeneration: Findings From a Time-and-Motion Study.* Am J Ophthalmol, 2015. **160**(4): p. 725-31 e1.
29. Keenan, T.D., C.J. Wotton, and M.J. Goldacre, *Trends over time and geographical variation in rates of intravitreal injections in England.* Br J Ophthalmol, 2012. **96**(3): p. 413-8.
30. Parikh, R., et al., *Trends of Anti-Vascular Endothelial Growth Factor Use in Ophthalmology Among Privately Insured and Medicare Advantage Patients.* Ophthalmology, 2016.
31. Cohen, D., *Why have UK doctors been deterred from prescribing Avastin?* BMJ, 2015. **350**: p. h1654.
32. Hutton, D., et al., *Switching to less expensive blindness drug could save medicare part B $18 billion over a ten-year period.* Health Aff (Millwood), 2014. **33**(6): p. 931-9.
33. Generics and biosimilars initiative. *Biosimilars of ranibizumab*. 2015 18 May 2017]; Available from: http://gabionline.net/Biosimilars/General/Biosimilars-of-ranibizumab.
34. Charlton, V., et al., *Cost effective but unaffordable: an emerging challenge for health systems.* BMJ, 2017. **356**: p. j1402.
35. Boulanger-Scemama, E., et al., *Ranibizumab for exudative age-related macular degeneration: A five year study of adherence to follow-up in a real-life setting.* J Fr Ophtalmol, 2015. **38**(7): p. 620-7.
36. Austeng, D., et al., *Nurse-administered intravitreal injections of anti-VEGF: study protocol for noninferiority randomized controlled trial of safety, cost and patient satisfaction.* BMC Ophthalmol, 2016. **16**(1): p. 169.
37. Chin-Yee, D., et al., *A systematic review of as needed versus treat and extend ranibizumab or bevacizumab treatment regimens for neovascular age-related macular degeneration.* Br J Ophthalmol, 2016. **100**(7): p. 914-917.
38. Violato, M., et al., *Cost-effectiveness of community versus hospital eye service follow-up for patients with quiescent treated age-related macular degeneration alongside the ECHoES randomised trial.* BMJ Open, 2016. **6**(10): p. e011121.
39. Neffendorf, J.E., et al., *StereoTactic radiotherapy for wet Age-Related macular degeneration (STAR): study protocol for a randomised controlled clinical trial.* Trials, 2016. **17**(1): p. 560.
40. Stukel, T.A., et al., *Standardized Rates of Recurrent Outcomes.* Statistics in Medicine, 1994. **13**(17): p. 1781-1791.

For peer review only - http://bmjopen.bmj.com/site/about/guidelines.xhtml

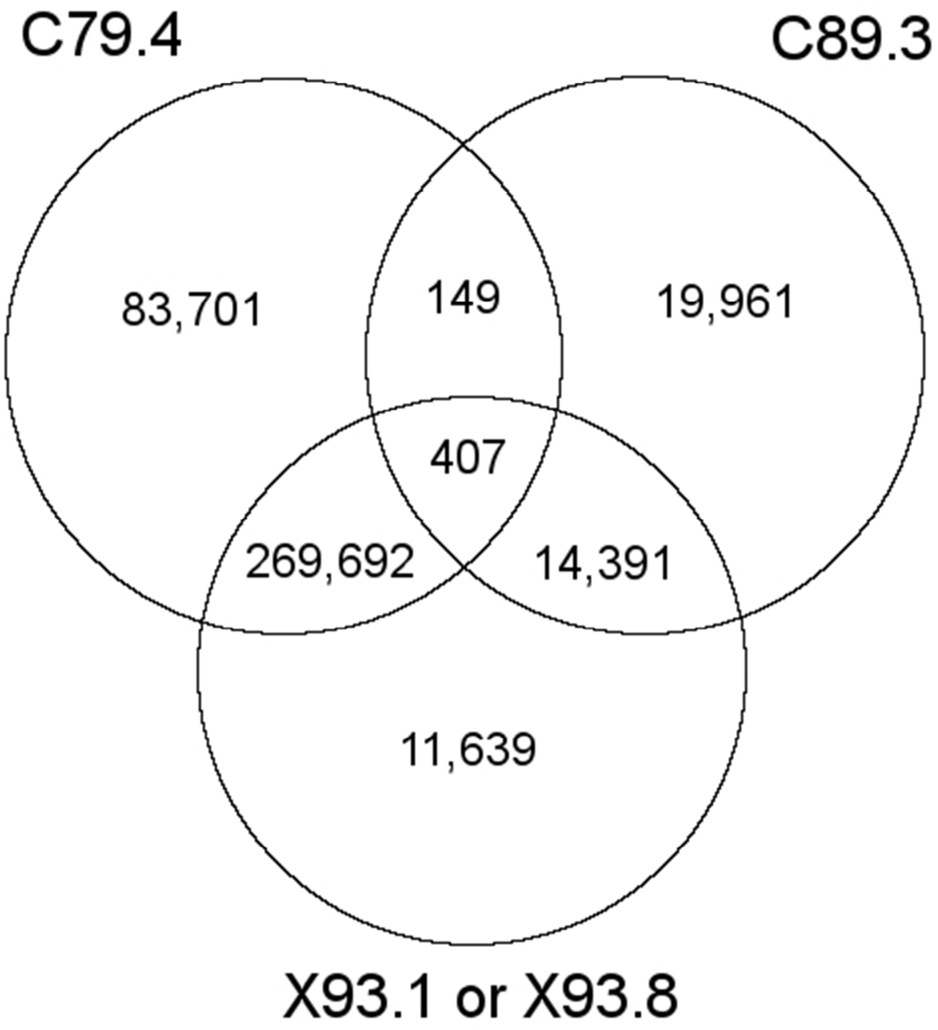

Figure 1 – OPCS codes used to identify anti-VEGF injections: 2014/15
Note: 2,479 eye injection procedures were excluded due to combination with X93.2 or X93.3 high-cost drug codes.

40x46mm (300 x 300 DPI)

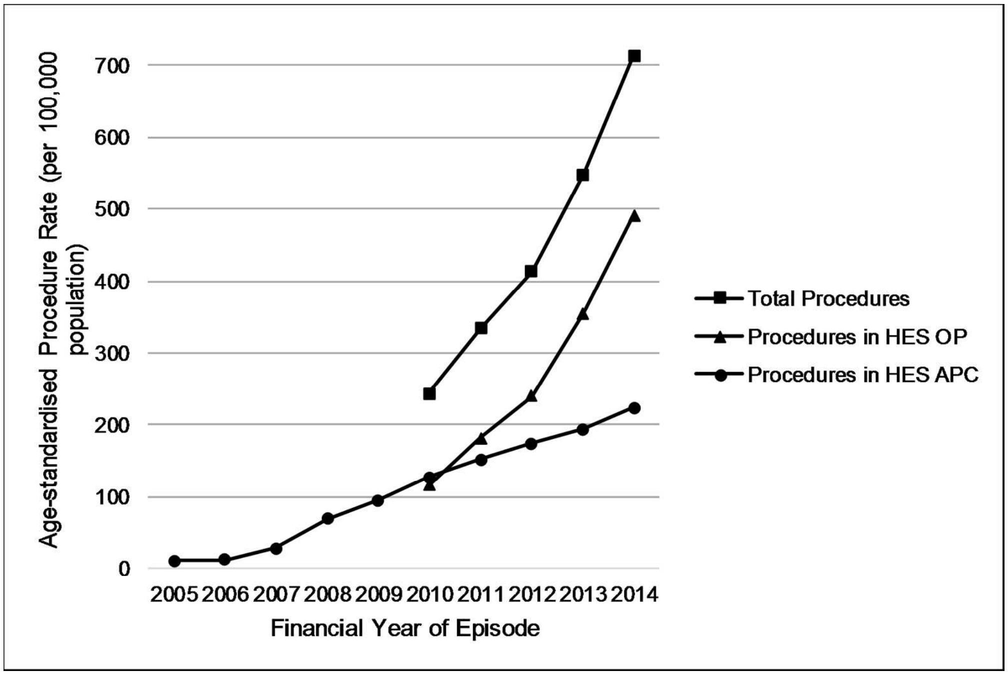

Figure 2. Directly-standardised intravitreal injection procedure rates, England, 2005/06-2014/15
Note: OP = Outpatient; APC = Admitted Patient Care

148x98mm (300 x 300 DPI)

For peer review only

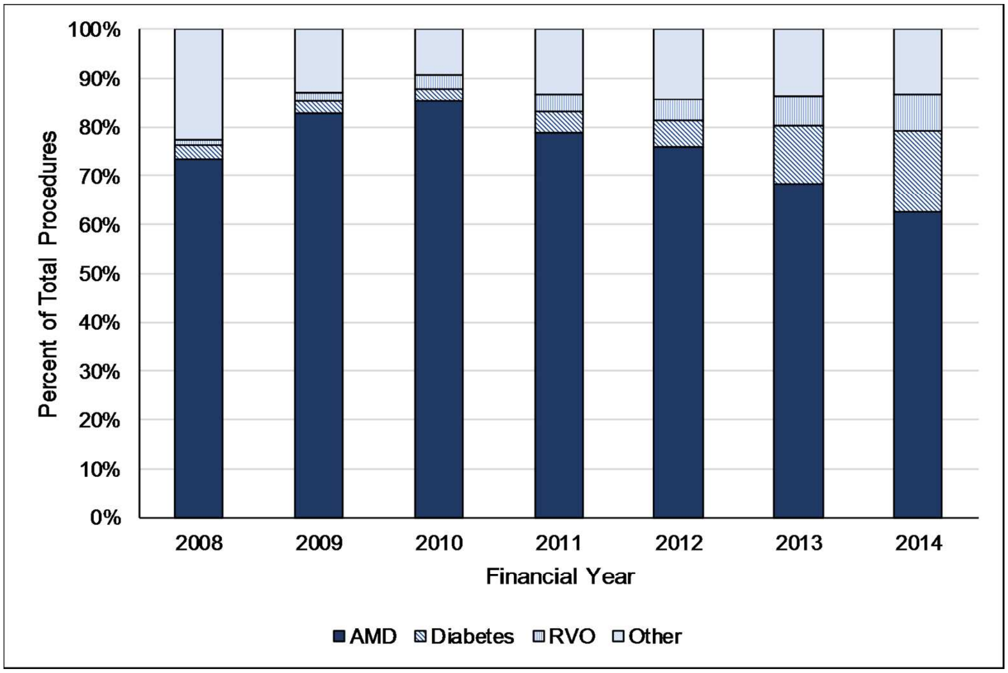

Figure 3. Percentage of total eye injection procedures in HES APC for different primary diagnoses, England, 2008/09-2014/15.
Note: nAMD = Neovascular Age-Related Macular Degeneration; DMO = Diabetic Macular Oedema; RVO = Retinal Vein Occlusion; Due to very small numbers of procedures for Myopic Choroidal Neo-Vascularisation (<=10 per year), this was included in 'Other'.

150x99mm (300 x 300 DPI)

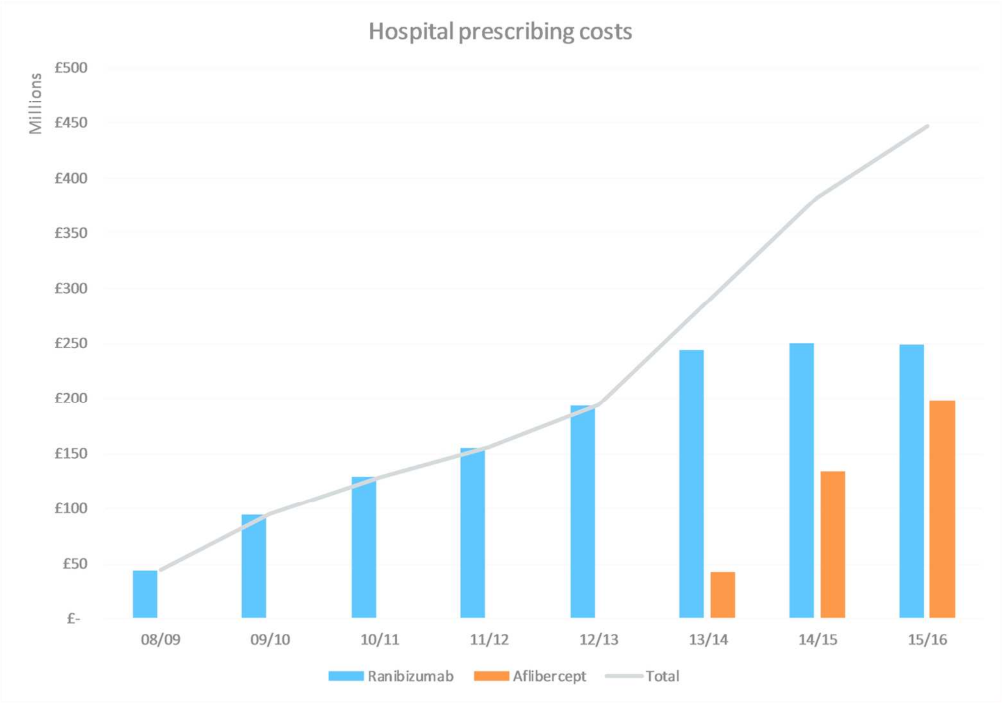

Figure 4. Hospital prescribing costs for Ranibizumab and Aflibercept, 2008/09 to 2015/16

136x95mm (300 x 300 DPI)

For peer review only

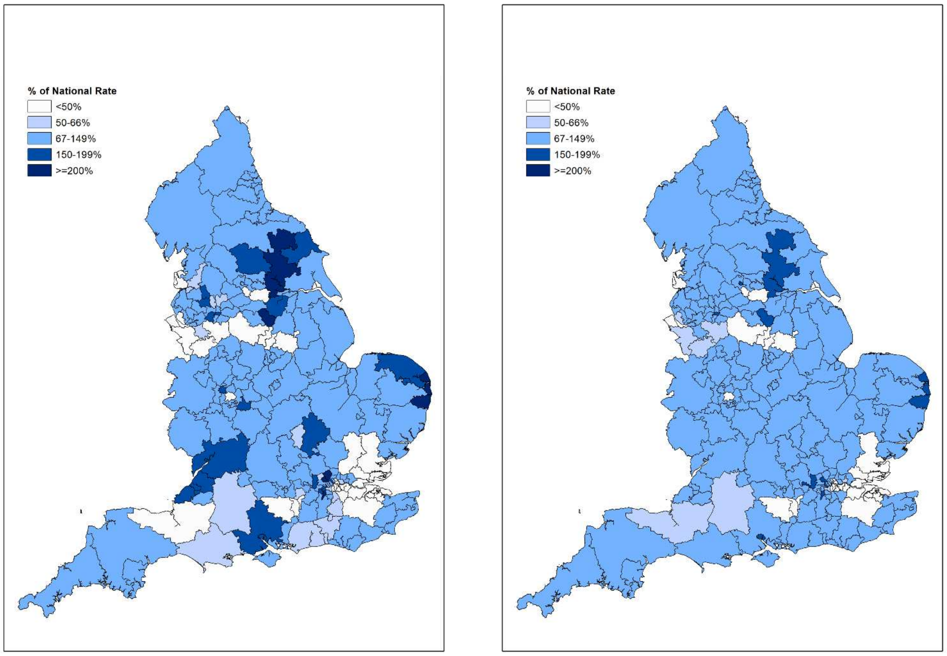

Figure 5a. Indirectly age-sex-deprivation-ethnicity standardised procedure ratios for combined eye injection procedures (OPCS-4: C794 and C893) by CCG in England, 2014/15.
Figure 5b. Indirectly age-sex-deprivation-ethnicity standardised patient ratios for combined eye injection procedures (OPCS-4: C794 and C893) by CCG in England, 2014/15.

420x296mm (300 x 300 DPI)

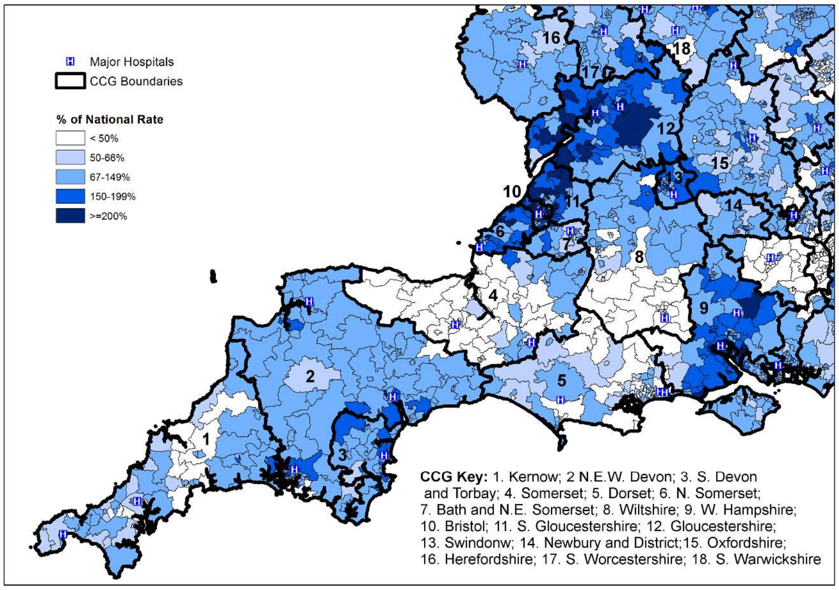

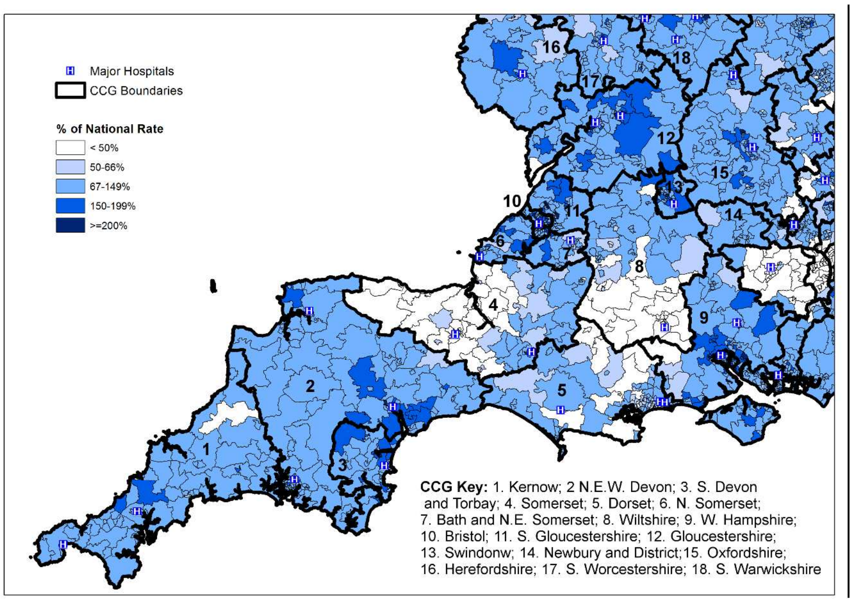

Figure 6a. Indirectly standardised procedure ratios for combined eye injection procedures by MSOA in SW England, 2014/15
Figure 6b. Indirectly standardised patient ratios for combined eye injection procedures by MSOA in SW England, 2014/15
Note: Major hospitals marked including those in NHS Trusts providing 100+ procedures.

296x414mm (300 x 300 DPI)

The RECORD statement – checklist of items, extended from the STROBE statement, that should be reported in observational studies using routinely collected health data.

	Item No.	STROBE items	Location in manuscript where items are reported	RECORD items	Location in manuscript where items are reported
**Title and abstract**					
	1	(a) Indicate the study's design with a commonly used term in the title or the abstract (b) Provide in the abstract an informative and balanced summary of what was done and what was found	P2, L6<br><br>P2, L6-27	RECORD 1.1: The type of data used should be specified in the title or abstract. When possible, the name of the databases used should be included.<br><br>RECORD 1.2: If applicable, the geographic region and timeframe within which the study took place should be reported in the title or abstract.<br><br>RECORD 1.3: If linkage between databases was conducted for the study, this should be clearly stated in the title or abstract.	P2, L6 & 7<br><br>P2, L6-10<br><br>N/A
**Introduction**					
Background rationale	2	Explain the scientific background and rationale for the investigation being reported	P4-5		
Objectives	3	State specific objectives, including any prespecified hypotheses	P5, L21-26		
**Methods**					
Study Design	4	Present key elements of study design early in the paper	P6-9		
Setting	5	Describe the setting, locations, and relevant dates, including periods of recruitment, exposure, follow-up, and data collection	P6, L2-24<br>P9, L4-13		
Participants	6	*(a) Cohort study* - Give the eligibility criteria, and the	P6, L12-24<br>P7, L1-18	RECORD 6.1: The methods of study population selection (such as codes or	P6, L12-24<br>P7, L1-18

For peer review only

		sources and methods of selection of participants. Describe methods of follow-up<br>*Case-control study* - Give the eligibility criteria, and the sources and methods of case ascertainment and control selection. Give the rationale for the choice of cases and controls<br>*Cross-sectional study* - Give the eligibility criteria, and the sources and methods of selection of participants<br><br>*(b) Cohort study* - For matched studies, give matching criteria and number of exposed and unexposed<br>*Case-control study* - For matched studies, give matching criteria and the number of controls per case	N/A	algorithms used to identify subjects) should be listed in detail. If this is not possible, an explanation should be provided.<br><br>RECORD 6.2: Any validation studies of the codes or algorithms used to select the population should be referenced. If validation was conducted for this study and not published elsewhere, detailed methods and results should be provided.<br><br>RECORD 6.3: If the study involved linkage of databases, consider use of a flow diagram or other graphical display to demonstrate the data linkage process, including the number of individuals with linked data at each stage.	P6, L12-17<br><br>N/A
Variables	7	Clearly define all outcomes, exposures, predictors, potential confounders, and effect modifiers. Give diagnostic criteria, if applicable.	P7, L20-24<br>P8, L1-18	RECORD 7.1: A complete list of codes and algorithms used to classify exposures, outcomes, confounders, and effect modifiers should be provided. If these cannot be reported, an explanation should be provided.	P7, L20-24<br>P8, L1-18
Data sources/ measurement	8	For each variable of interest, give sources of data and details of methods of assessment (measurement).<br>Describe comparability of assessment methods if there is more than one group	P7 – P9		
Bias	9	Describe any efforts to address potential sources of bias	P7, L20-24<br>P8, L1-18		
Study size	10	Explain how the study size was	N/A – there was no		

For peer review only

		arrived at	sample size calculation		
Quantitative variables	11	Explain how quantitative variables were handled in the analyses. If applicable, describe which groupings were chosen, and why	P7, L20-24<br>P8, L1-18		
Statistical methods	12	(a) Describe all statistical methods, including those used to control for confounding<br>(b) Describe any methods used to examine subgroups and interactions<br>(c) Explain how missing data were addressed<br>(d) *Cohort study* - If applicable, explain how loss to follow-up was addressed<br>*Case-control study* - If applicable, explain how matching of cases and controls was addressed<br>*Cross-sectional study* - If applicable, describe analytical methods taking account of sampling strategy<br>(e) Describe any sensitivity analyses	P7, L20-24<br>P8, L1-18<br><br>N/A<br><br>N/A<br><br>N/A		
Data access and cleaning methods		..		RECORD 12.1: Authors should describe the extent to which the investigators had access to the database population used to create the study population.<br><br>RECORD 12.2: Authors should provide information on the data cleaning methods used in the study.	P6, L1-11

For peer review only

				P7, L20-24<br>P8, L1-18	
Linkage		..		RECORD 12.3: State whether the study included person-level, institutional-level, or other data linkage across two or more databases. The methods of linkage and methods of linkage quality evaluation should be provided.	N/A
**Results**					
Participants	13	(a) Report the numbers of individuals at each stage of the study (*e.g.*, numbers potentially eligible, examined for eligibility, confirmed eligible, included in the study, completing follow-up, and analysed)<br>(b) Give reasons for non-participation at each stage.<br>(c) Consider use of a flow diagram	Figure 1, P10, L1-7	RECORD 13.1: Describe in detail the selection of the persons included in the study (*i.e.,* study population selection) including filtering based on data quality, data availability and linkage. The selection of included persons can be described in the text and/or by means of the study flow diagram.	Figure 1
Descriptive data	14	(a) Give characteristics of study participants (*e.g.*, demographic, clinical, social) and information on exposures and potential confounders<br>(b) Indicate the number of participants with missing data for each variable of interest<br>(c) *Cohort study* - summarise follow-up time (*e.g.*, average and total amount)	Table 2<br><br>N/A		
Outcome data	15	*Cohort study* - Report numbers of outcome events or summary measures over time<br>*Case-control study* - Report numbers in each exposure category, or summary measures of exposure	Figures 2-6		

For peer review only

		Cross-sectional study - Report numbers of outcome events or summary measures			
Main results	16	(a) Give unadjusted estimates and, if applicable, confounder-adjusted estimates and their precision (e.g., 95% confidence interval). Make clear which confounders were adjusted for and why they were included<br>(b) Report category boundaries when continuous variables were categorized<br>(c) If relevant, consider translating estimates of relative risk into absolute risk for a meaningful time period	Table 2<br>N/A<br>N/A		
Other analyses	17	Report other analyses done—e.g., analyses of subgroups and interactions, and sensitivity analyses	N/A		
**Discussion**					
Key results	18	Summarise key results with reference to study objectives	P12, L1-9		
Limitations	19	Discuss limitations of the study, taking into account sources of potential bias or imprecision. Discuss both direction and magnitude of any potential bias	P12 L11-24<br>P13, L1-4	RECORD 19.1: Discuss the implications of using data that were not created or collected to answer the specific research question(s). Include discussion of misclassification bias, unmeasured confounding, missing data, and changing eligibility over time, as they pertain to the study being reported.	P12 L11-24<br>P13, L1-4
Interpretation	20	Give a cautious overall interpretation of results considering objectives, limitations, multiplicity of analyses, results from similar studies, and other relevant	P15, L20-23<br>P16		

For peer review only

		evidence			
Generalisability	21	Discuss the generalisability (external validity) of the study results	P13, L9-25		
**Other Information**					
Funding	22	Give the source of funding and the role of the funders for the present study and, if applicable, for the original study on which the present article is based	P17		
Accessibility of protocol, raw data, and programming code		..		RECORD 22.1: Authors should provide information on how to access any supplemental information such as the study protocol, raw data, or programming code.	N/A

*Reference: Benchimol EI, Smeeth L, Guttmann A, Harron K, Moher D, Petersen I, Sørensen HT, von Elm E, Langan SM, the RECORD Working Committee. The REporting of studies Conducted using Observational Routinely-collected health Data (RECORD) Statement. *PLoS Medicine* 2015; in press.

*Checklist is protected under Creative Commons Attribution (CC BY) license.

For peer review only
